# Multiphasic Organization and Differential Dynamics of Proteins within Protein–DNA Biomolecular Condensates

**DOI:** 10.1021/acs.jpcb.5c03987

**Published:** 2025-09-15

**Authors:** Ashish Shyam Tangade, Anupam Mondal, Jiahui Wang, Young C. Kim, Jeetain Mittal

**Affiliations:** † Artie McFerrin Department of Chemical Engineering, 14736 Texas A&M University, College Station, Texas 77843, United States; ‡ Center for Materials Physics and Technology, 41487 Naval Research Laboratory, Washington, District of Columbia 20375, United States; § Department of Chemistry, 14736 Texas A&M University, College Station, Texas 77843, United States; ∥ Interdisciplinary Graduate Program in Genetics and Genomics, 14736 Texas A&M University, College Station, Texas 77843, United States

## Abstract

Biomolecular condensates formed through liquid–liquid phase separation are increasingly recognized as critical regulators of genome organization and gene expression. While the role of proteins in driving phase separation is well-established, how DNA modulates the structure and dynamics of protein–DNA condensates remains less well understood. Here, we employ a minimalist coarse-grained model to investigate the interplay between homotypic protein–protein and heterotypic protein–DNA interactions in governing condensate formation, composition, and internal dynamics. Our simulations reveal that DNA chain length and flexibility critically influence condensate morphology, leading to the emergence of multiphasic and core–shell organizations under strong heterotypic interactions. We find that DNA recruitment into the condensate significantly alters protein mobility, giving rise to differential dynamics of proteins within the condensate. By analyzing the distribution profiles of protein displacements, we identify up to five distinct diffusion modes, including proteins bound to DNA, confined within the dense phase, or freely diffusing. These results provide a mechanistic framework for interpreting spatially heterogeneous protein dynamics observed in chromatin condensates and emphasize the direct role of DNA in tuning condensate properties. Our findings provide new insights into how biophysical parameters may control the functional architecture of protein–DNA condensates in biological systems.

## Graphical abstract

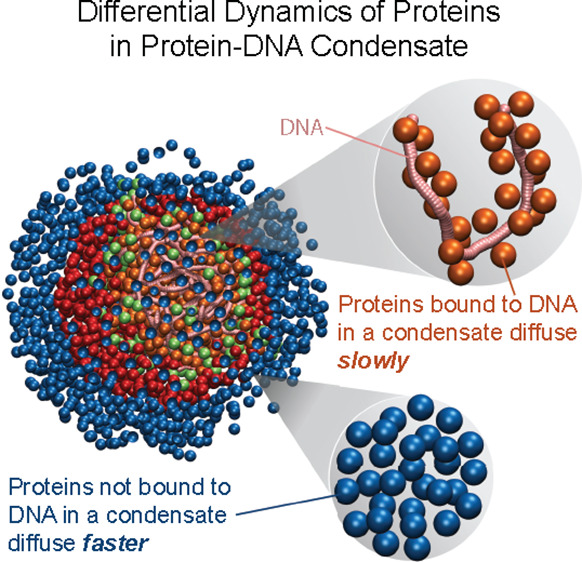

## Introduction

Biomolecular condensates (BMCs), also known as membraneless organelles, are dynamic assemblies in cells formed via liquid–liquid phase separation (LLPS) of proteins and nucleic acids. These condensates lack a surrounding membrane yet maintain a localized high concentration of specific biomolecules that facilitates diverse biological functions.^1,2^ A few common examples of BMCs include nucleoli, stress granules, paraspeckles, Cajal bodies, and mitochondrial nucleoids.^3,4^ BMCs are known to play a crucial role in genome organization, transcriptional regulation, RNA processing, cell signaling, and many other cellular functions.^5–9^ The assembly and internal organization of BMCs are primarily governed by homotypic interactions between proteins, heterotypic interactions between proteins and nucleic acids, as well as among nucleic acids themselves. These interactions arise from a combination of electrostatics, hydrogen bonding, π–π stacking, cation−π interactions, and hydrophobic effects^10–14^ whose balance determines not only whether phase separation occurs, but also the internal structure, morphology, dynamics, and functional compartmentalization within the condensates.

Several experimental and computational studies have probed the role of protein–DNA interactions in driving phase separation.^15–25^ For instance, the protein Fused-in-Sarcoma (FUS), which comprises both prion-like and RNA/DNA-binding domains, undergoes phase separation with single- and double-stranded DNA,^17^ revealing that electrostatic and π-stacking interactions between FUS and nucleic acids can drive heterotypic condensation.^19,26,27^ Similarly, the transcription factor Forkhead box protein A1 (FoxA1) forms condensates with double-stranded DNA and can spatially organize distal DNA regions into close proximity to carry out cellular functions.^24^ Heterochromatin protein 1 (HP1α) is another example that has been shown to compact DNA and form condensates, possibly due to homotypic HP1α-HP1α oligomerization.^28^

In addition to these molecular interactions, a few other experimental studies^28–30^ have recently shown that the physical characteristics of DNA such as its length and flexibility, play critical roles in modulating the phase behavior of biomolecular condensates. For example, Ryu et al.^29^ demonstrated that the formation of protein–DNA clusters by structural maintenance of chromosome (SMC) proteins exhibits a critical dependence on DNA length: for shorter DNA chains, the cluster size remains largely insensitive to DNA length, while for longer DNA chains, the cluster size increases strongly with DNA length, thereby promoting phase separation. Likewise, Shakya and King^30^ showed that flexible DNA leads to denser and more compact coacervates with poly-l-lysine, indicating that DNA stiffness influences condensate architecture and internal mobility.

Computational studies using coarse-grained (CG) models^31,32^ have been powerful in exploring protein–DNA condensates across large system sizes and time scales that are inaccessible to all-atom molecular dynamics. Using such CG models, recently it has been shown that HP1α and histone H1 proteins can form multiphasic condensates with layered organization that is mediated by DNA.^33^ Ancona and Brackley^34^ used simple CG simulations to map the phase behavior of HP1α–chromatin systems under different interaction regimes, revealing that the relative strength of homotypic versus heterotypic interactions governs phase stability and internal organization. Multiscale simulations have further shown how post-translational modifications, such as phosphorylation of HP1α, alter its interaction with DNA and modulate condensate formation and physical properties.^22,23^ Related computational work on protein–RNA systems has demonstrated that differences in interaction strengths can give rise to layered condensate organization,^35,36^ providing a useful conceptual framework for understanding analogous multiphasic behavior in protein–DNA condensates. Despite these advances, a detailed mechanistic understanding of how homotypic and heterotypic interactions interplay with DNA’s physical properties to determine condensate architecture and protein dynamics remains incomplete.

To address this gap, we develop a minimalistic coarse-grained model to disentangle the roles of homotypic and heterotypic interactions, and DNA physical properties, in the formation and organization of protein–DNA condensates. In our model, protein molecules are treated as single beads, while DNA molecules are modeled as polymer chains. By varying protein–protein (homotypic) and protein–DNA (heterotypic) interaction strengths, along with different physical properties of DNA chains, we systematically map the phase behavior and structural organization of the resulting condensates. Our results reveal a rich landscape of condensate behavior. We identify regimes where homotypic interactions alone are insufficient to induce phase separation, but heterotypic interactions with DNA enable condensate formation. Strikingly, when both interaction types are present, condensates adopt a multiphasic architecture, characterized by DNA-bound protein-rich cores surrounded by a protein-enriched shell. Furthermore, we show that DNA length and flexibility critically modulate the phase separation and the internal organization of condensates. Importantly, we uncover distinct dynamical modes of proteins within condensates, including freely diffusing, transiently DNA-bound, and stably bound populations, suggesting spatially heterogeneous molecular mobility. Collectively, our results reveal how the interplay between interaction types and DNA physical features shapes the structure, composition, and dynamic behavior of protein–DNA condensates, providing mechanistic insight into principles underlying nuclear organization.

## Methods

Simulating large-scale phenomena such as LLPS requires a careful balance of computational expense with system size. In this work, we develop a minimalistic coarse-grained (CG) model of protein and DNA and their interactions to study the phase separation of biomolecular condensates. Similar CG models have been widely used to study intrinsically disordered proteins and their phase behavior,^37–42^ as well as protein–DNA complexes and chromatin organization with great success.^22,33,34,43^

### Protein Model

The protein molecule is modeled as a single spherical bead, as schematically shown in Figure 1A. The size of the protein bead is considered based on the radius of gyration (*R*_g_) of DNA-binding proteins typically implicated in biomolecular condensates. Specifically, we set the diameter of the protein bead to σ_P_ = 50 Å, which approximates twice the average *R*_g_ value for proteins such as TFAM and HP1α.^22,44^

**Figure 1:**
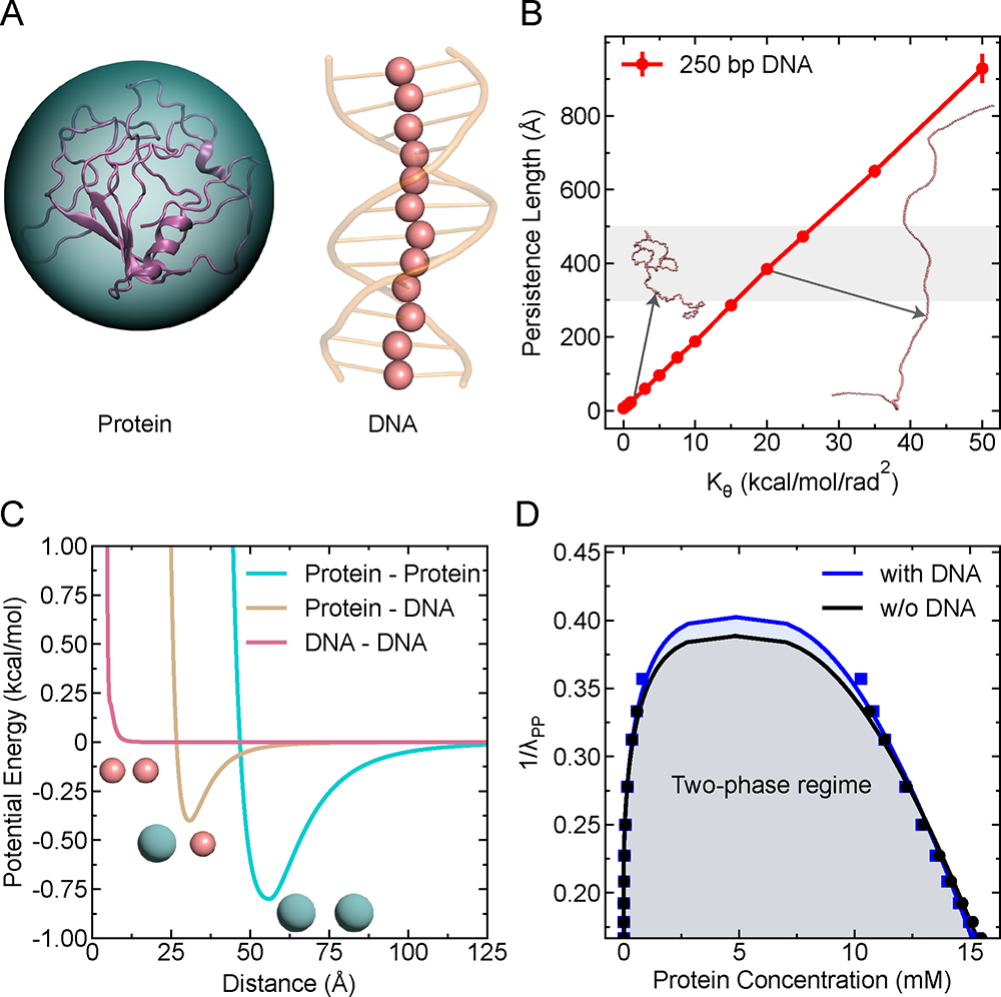
Description of the minimalistic model. (A) Schematic of the CG model: protein is modeled as a single spherical bead, and DNA as bead–spring polymers. (B) Persistence length of the polymeric DNA of length 250 bp as a function of the angle potential parameter *K*_θ_. The shaded region indicates the experimentally observed range of persistence length for dsDNA. Representative DNA conformations are shown for two *K*_θ_ values. (C) Ashbaugh-Hatch potential energy for protein–protein (homotypic), protein–DNA (heterotypic), and DNA–DNA interactions as a function of interbead distance. (D) Phase diagrams showing protein phase separation as a function of homotypic interaction strength λ_PP_, in the absence and presence of DNA (λ_PD_ = 0.5, DNA length *L* = 250 bp), exhibiting phase coexistence.

The mass of each protein bead is chosen as 5000 g/mol. Although naturally occurring DNA-binding proteins like HP1α typically weigh ∼22 to 25 kDa (22,000–25,000 g/mol), assigning that mass to a coarse-grained bead significantly slows diffusive motion in the simulation, resulting in a substantially longer simulation time to reach equilibrium. Thus, we adopted a reduced value of mass to improve computational efficiency and maintain a balance between the protein and DNA bead masses.

### DNA Model

The DNA molecule is modeled as a polymeric chain in which each monomer corresponds to one base pair (bp) for double-stranded DNA and/or one nucleotide for single-stranded DNA, as schematically illustrated in Figure 1A. The total potential energy of the DNA chain comprises bond stretching and angular bending contributions, as given by

$$U_{\mathrm{DNA}}=U_{\mathrm{bond}}+U_{\mathrm{angle}}$$ 1

The bond energy between successive beads is given by a harmonic potential:

$$U_{\mathrm{bond}}=K_{b}(r-r_{0}{)}^{2}$$ 2

where *K*_b_ = 20 kcal/mol/Å^2^ is the spring constant and *r*_0_ = 5.5 Å is the equilibrium bond length.

To modulate the bending rigidity of DNA, the angular potential between three consecutive monomers is defined as

$$U_{\mathrm{angle}}=K_{\theta }(\theta -\theta_{0}{)}^{2}$$ 3

where *K*_θ_ is the spring constant for the angle potential and θ_0_ = 180° is the equilibrium angle between two successive bonds of DNA. In order to determine the flexibility of the DNA chain ideal for double-stranded DNA (dsDNA), we varied *K*_θ_ values and measured the persistence length (see Supporting Information for calculation details) of a single DNA chain of length 250 bp as shown in Figure 1B. The experimentally^45,46^ reported values of persistence length for dsDNA are highlighted with gray shaded region, based on which we set the *K*_θ_ value to 20 kcal/mol/rad^2^ that qualitatively replicates the flexibility of dsDNA. Additionally, the diameter (σ_D_) and mass of each monomer are chosen as 5 Å and 500 g/mol, respectively.

### Nonbonded Interactions

For modeling the effective nonbonded interactions among proteins, protein–DNA, and DNA, we used a modified Lennard-Jones (LJ) potential,^37,47^ where attractive interactions between two particles are scaled independently of short-range repulsive interactions. The nonbonded interactions are modeled by the following Ashbaugh-Hatch^47^ potential

$$U_{\mathrm{nb}}(r)=\{\begin{array}{ll} U_{\mathrm{LJ}}(r)+(1-\lambda_{ij})\epsilon , & \mathrm{if}\;r\leq 2^{1/6}\sigma \\ \lambda_{ij}U_{\mathrm{LJ}}(r), & \mathrm{otherwise} \end{array}$$ 4

in which *U*_LJ_(*r*) is the standard Lennard-Jones potential,

$$U_{\mathrm{LJ}}(r)=4\epsilon [(\frac{\sigma }{r}{)}^{12}-(\frac{\sigma }{r}{)}^{6}]$$ 5

where ϵ is the interaction strength fixed to a value of 0.2 kcal/mol and λ_*ij*_ is the hydropathy between two interacting species. The arithmetic average is set as the combination rule for the size σ (i.e., σ_PD_ = (σ_P_ + σ_D_)/2). The strength of homotypic and heterotypic interactions can be varied by changing the hydropathy value λ (Figure 1C), while the interactions between all DNA beads are made repulsive by setting λ_DD_ = −1, with the exception of the directly bonded DNA beads.

To relate protein–DNA interaction strength (λ_PD_) with the experimentally relevant protein–DNA binding affinities, we calculated the dissociation constants (*K*_D_) for protein–DNA binding with varying heterotypic interaction strengths (Figure S1, see Supporting Information for calculation details). The resulting *K*_D_ values were then compared with the experimentally reported values for FUS–dsDNA^48^ and HP1α–dsDNA binding^28^ (Figure S1). These comparisons indicate that the calculated *K*_D_ values are generally consistent with experimental measurements, with many lying directly within the reported experimental ranges and others deviating by less than an order of magnitude. This indicates that our chosen interaction strengths are physically reasonable for biological systems.

### Simulation Protocol

To explore the phase behavior of protein–DNA mixtures, we simulated systems with a fixed stoichiometric ratio of protein to DNA monomers of 1:1. Specifically, we considered 5000 CG protein beads and a number of DNA chains of a given length *L* base pairs such that the total number of DNA beads also equaled 5000. All particles were initially placed randomly at the center of a cubic box with an edge length of 2000 Å with periodic boundary conditions in all directions. We performed Langevin dynamics simulations at a fixed temperature of 300 K, with the friction coefficient γ_*i*_ = *m*_*i*_/*t*_damp_, where *m*_*i*_ is the mass of the *i*th particle and *t*_damp_ is the damping factor that is set to 1000 ps. The equations of motion were integrated using a velocity-Verlet algorithm with a time step of 10 fs. All simulations were performed using the HOOMD-blue molecular dynamics engine (version 2.9.3),^49,50^ utilizing the additional features provided by azplugins (version 0.11.0).

## Results and Discussion

To illustrate the effectiveness of our minimal model in capturing biomolecular condensate formation, we first examined whether it could reproduce phase separation in systems with only proteins and in mixed systems containing both proteins and DNA. For this, we varied the interaction strength λ_PP_ between proteins in the absence and presence of DNA and performed 500 ns simulations for each λ_PP_ value. We divided the simulation trajectory into five equal blocks in which the first block (i.e., initial 20% of the data) was discarded for equilibration and computed the mean and standard error by calculating the block average from the remaining data. As shown in Figure 1D, both systems exhibit clear signatures of coexistence of two phases, characterized by the emergence of a dense condensate coexisting with a surrounding dilute phase, in which most DNA chains are recruited into the condensate, while lower fraction of proteins stay in the dilute phase for the protein–DNA system. This result suggests that our model is well suited to study condensate formation and gives us the confidence to use it further in dissecting how homotypic (protein–protein) and heterotypic (protein–DNA) interactions influence the phase behavior of protein–DNA condensates.

### Phase Behavior of Protein–DNA Condensates as a Function of Homotypic and Heterotypic Interactions

To delineate the role of homotypic and heterotypic interactions in the formation and composition of protein–DNA condensates, we systematically varied the homotypic (λ_PP_) and heterotypic (λ_PD_) interaction strengths across a broad range of parameter space (Figure 2). For these simulations, we used DNA chains of length 250 bp, with 20 such chains present in the system. We performed a 2 μs long simulation for each combination of λ_PP_ and λ_PD_. To assess equilibration, we monitored the dilute-phase protein concentration as a function of simulation time as shown in Figure S2. The data exhibited initial fluctuations and then plateaued, indicating that the system reached equilibrium. Consequently, we determined that the initial 400 ns (20% of the trajectory) corresponded to the equilibration period and the remaining 1.6 μs of data was used for production analysis. To quantify condensate formation, we calculated the probability distribution of the size of clusters formed during the simulation. For identifying a cluster, we used a distance criterion where molecules within 1.5 σ_PD_ are considered as a part of the same cluster using the Freud^51^ Python library (version 2.12.1). As shown in Figure S3, we observed that the largest cluster was significantly dominant in size compared to the other clusters, so we chose to use the largest cluster for quantifying condensate properties. For that, we evaluated the protein fraction (Figure 2A) and DNA fraction (Figure 2B) within the largest cluster which are defined as

$$f_{P}=\frac{N_{P}^{\mathrm{cluster}}}{N_{P}^{\mathrm{total}}},f_{D}=\frac{N_{D}^{\mathrm{cluster}}}{N_{D}^{\mathrm{total}}}$$

where *N*_P_^cluster^ and *N*_D_^cluster^ are the numbers of protein and DNA beads, respectively, in the largest cluster, and *N*_P_^total^ and *N*_D_^total^ are the total numbers of protein and DNA beads in the system. These fractions provide insight into both the size and composition of the condensates (whether they are protein-rich, DNA-rich, or mixed protein–DNA) relative to the dilute phase and directly reflect how the interaction strengths drive phase separation and recruitment of each component within condensates.

**Figure 2:**
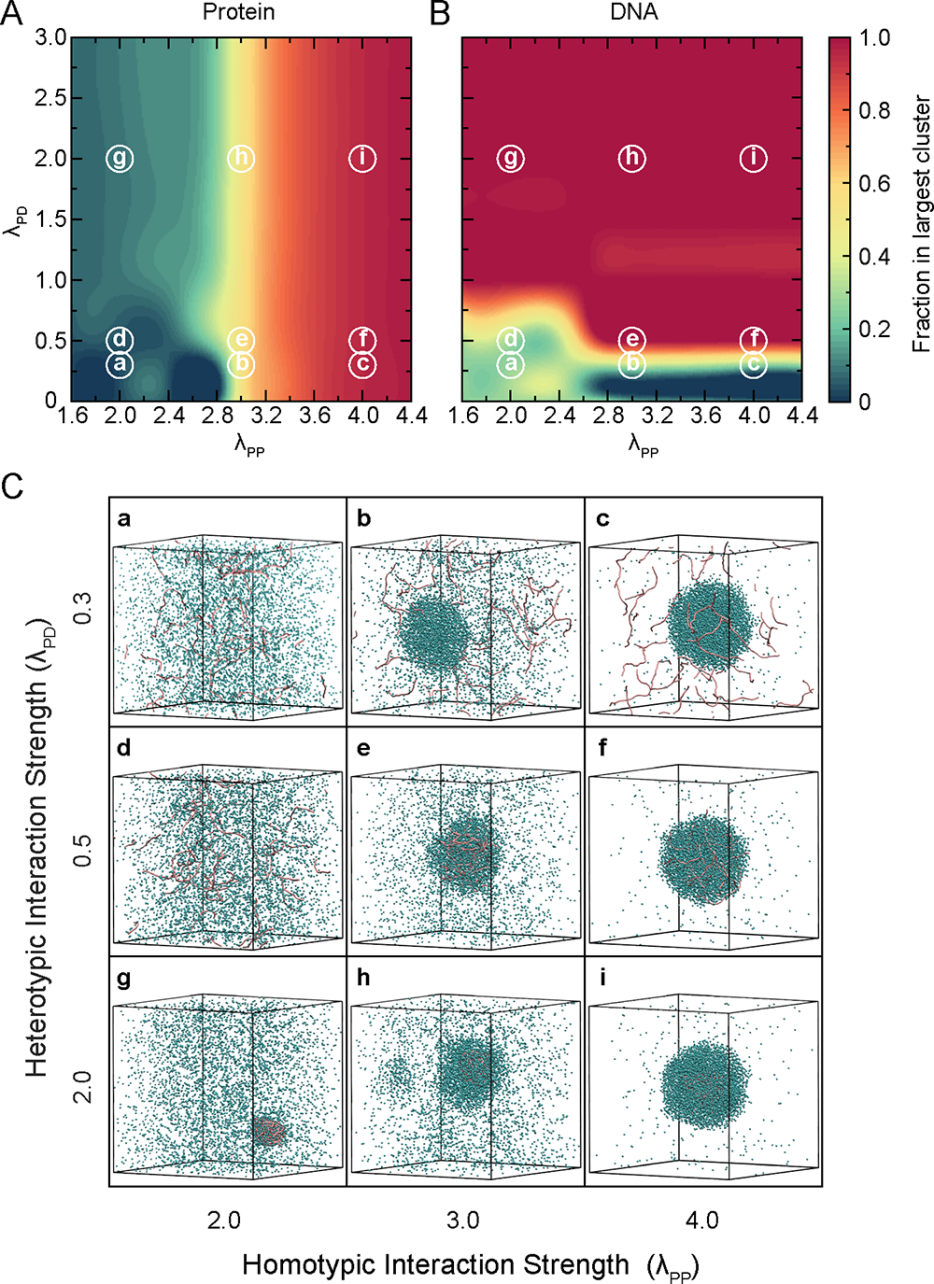
Phase space of homotypic and heterotypic interactions. Heatmap showing the average fraction of (A) protein and (B) DNA in the largest cluster for varying homotypic (λ_PP_) and heterotypic (λ_PD_) interaction strengths. (C) Representative snapshots from simulation for different regimes of homotypic and heterotypic interactions, labeled (a–i). Small letters circled in (A) and (B) correspond to the respective subfigures shown in (C). Proteins are shown as cyan beads whereas DNA molecules are represented by pink colored chains. Each snapshot illustrates distinct condensate compositions: (a, d) no condensation, (b, c) protein-rich condensates with DNA at periphery of condensate, (e, f) mixed protein–DNA condensates with fluid-like DNA, (g) DNA-scaffolded protein clusters via bridging, and (h, i) dense protein–DNA co-condensates with highly localized DNA cores. DNA chains of length 250 bp (20 in total) and 5000 protein beads are used in these simulations.

The resulting phase diagrams (Figure 2A,B) reveal a diverse landscape of condensate behaviors, characterized by distinct regimes determined by the interplay between homotypic and heterotypic interactions. For instance, when the homotypic interactions are strong (λ_PP_ ≥ 3) and the heterotypic interactions are weak (λ_PD_ < 0.5), we observe protein-rich condensates in which proteins undergo phase separation largely independent of DNA. In this regime, proteins dominate the largest cluster (Figure 2C, insets b,c), while DNA molecules remain mainly dispersed in the dilute phase. The small but nonzero DNA fraction (0.2–0.4) in the largest cluster arises because a DNA chain is counted as part of the cluster if any of its beads are in contact with it—typically when DNA segments extend into the condensate periphery. This organization, where proteins phase separate independently of DNA, is also observed in in vitro experiments of FUS protein, where the low complexity domain of FUS is responsible for interactions causing phase separation.^17,52,53^ With protein DEAD-box helicase 4 (Ddx4), a similar arrangement of components is observed where protein forms a condensate via homotypic interactions but does not recruit dsDNA into the condensate.^54^ In contrast, when both the homotypic and heterotypic interaction strengths are weak (λ_PP_ < 3 and λ_PD_ < 0.5), neither proteins nor DNA undergo phase separation, and the system remains homogeneous with all components remaining dispersed in the dilute phase (Figure 2C, inset a). This regime represents a baseline state where interaction strengths are insufficient to drive condensate formation. As the heterotypic interaction strength increases to a moderate range (0.5 ≤ λ_PD_ ≤ 1.0) while maintaining strong homotypic interactions, both proteins and DNA are recruited into the condensate, forming a mixed protein–DNA co-condensate. In this regime, the DNA remains fluid-like and dispersed across the entire condensate (Figure 2C, insets e,f). This organization of fluidized DNA can be of functional relevance for the transcriptional condensates, where the DNA needs to be accessible to transcriptional machinery, enhancers and coactivators.^55^ Interestingly, when heterotypic interactions are strong (λ_PD_ > 1.0) but homotypic interactions are weak (λ_PP_ < 3), DNA molecules serve as scaffolds and proteins alone do not phase separate but instead become localized by binding to DNA, forming “bridging” contacts between different DNA segments. This results in localized protein clusters around DNA with a relatively low protein fraction in the overall condensate (Figure 2C, inset g). This condensate formation is similar to the bridging induced phase separation, which has been proposed as another mechanism for phase separation of protein–DNA condensates^21,43,56^ and has also been experimentally reported in condensates formed with cohesion-SMC protein complexes^29^ and DNA-binding proteins from starved cells.^57^ Furthermore, when both homotypic and heterotypic interactions are strong, proteins undergo phase separation and simultaneously form bridging contacts with DNA. This leads to compact, dense co-condensates where DNA is sequestered in a highly localized region near the center of the droplet (Figure 2C, insets h,i). Such an arrangement within condensates is like the ones reported for the HP1α-DNA system, where proteins are dynamic but the DNA molecules are localized within a smaller region in the condensate.^28^ It also aligns with the hierarchical condensation observed in the protein Krüppel-like factor 4 (Klf4) on DNA, where Klf4 forms an adsorbed layer on DNA at lower protein concentrations and these adsorbed layers further form thicker condensates at higher protein concentrations.^58^

Based on these observations, we categorize the heterotypic interaction strength into three regimes: weak (λ_PD_ < 0.5), moderate (0.5 ≤ λ_PD_ ≤ 1.0), and strong (λ_PD_ > 1.0), and the homotypic interaction into two regimes: weak (λ_PP_ < 3) and strong (λ_PP_ ≥ 3). This classification helps us to further evaluate the impact of DNA physical features for modulating condensate properties within each interaction regime.

### Role of Heterotypic Interactions in the Organization of Protein–DNA Condensates

We next explored how homotypic and heterotypic interactions govern the internal organization of the components within the condensate. Specifically, we focused on two key aspects: the spatial configuration of DNA chains and the extent of protein binding to DNA, both of which are critical for understanding the structural and functional implications of condensate formation.

To probe DNA organization within the condensate, we computed the average radius of gyration (*R*_g_) of DNA molecules (see Supporting Information for calculation details) as a function of protein–DNA interaction strength (λ_PD_), for three different homotypic protein–protein interaction strengths (λ_PP_ = 2.0, 3.0, 4.0). As shown in Figure 3A, when protein–DNA interaction strength is weak (λ_PD_ < 0.5), the DNA chains remain extended, exhibiting an average *R*_g_ of approximately 295 Å, which is comparable to the value obtained for an isolated DNA chain of the same length, as indicated by the black dashed line. This suggests that under weak protein–DNA interactions, DNA adopts a relaxed conformation. As λ_PD_ increases, DNA becomes increasingly compact, with *R*_g_ dropping to as low as 100–125 Å. This compaction is most pronounced when the protein–protein interaction is weak (λ_PP_ = 2.0), meaning proteins cannot phase separate on their own and instead use DNA as a scaffold to assemble. This compaction behavior closely resembles that of protamine proteins, which causes extreme DNA compaction in sperm cells potentially rendering DNA transcriptionally inactive.^59^ Interestingly, dephosphorylation of protamine,^60^ which increases its interaction strength with DNA, shows even higher DNA compaction which is in accordance with the trend observed in our result. However, as we increase the homotypic interactions to λ_PP_ = 3.0 and 4.0, the DNA becomes less compact (larger *R*_g_) for the same λ_PD_. This suggests a competition between protein–protein and protein–DNA interactions: when proteins strongly interact with each other, fewer proteins are available to bind and compact DNA. As a result, DNA adopts a more expanded configuration. Such an expansion of DNA in the presence of strong protein–protein interactions might play a significant role in the accessibility of DNA to transcription assemblies, cofactors, and activators which then helps DNA to perform its function with these additional assemblies.^18,55,61,62^

**Figure 3:**
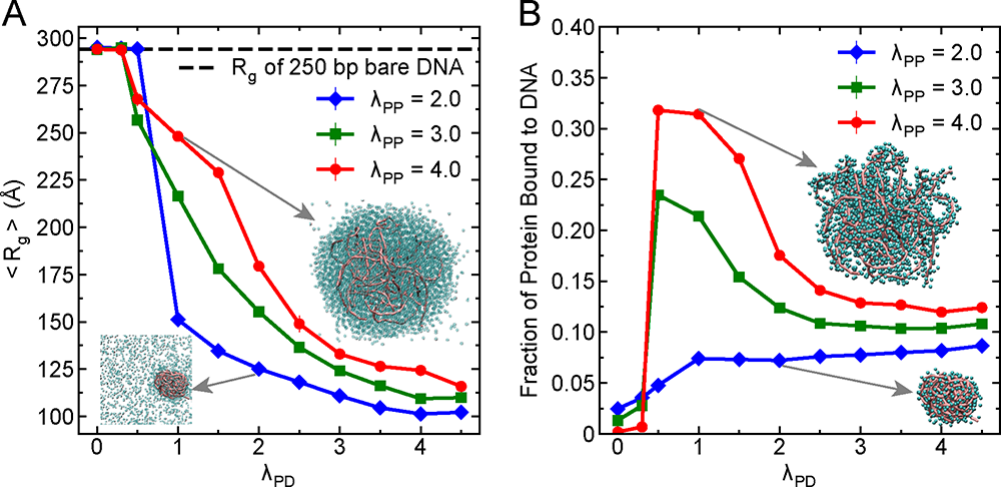
Heterotypic interactions affect the DNA compaction and protein bound to DNA. (A) Average radius of gyration (*R*_g_) of 250 bp DNA chains as a function of heterotypic protein–DNA interaction strength (λ_PD_) for three different homotypic protein–protein interaction strengths λ_PP_. The horizontal dashed line indicates the *R*_g_ of a single DNA chain of the same length of 250 bp in the absence of proteins. Two representative snapshots (insets) illustrate the structural organization of DNA within the condensate under weak and strong homotypic interactions. (B) Fraction of proteins bound to DNA as a function of λ_PD_, for the same three values of λ_PP_. Two representative snapshots (insets) show protein binding patterns in the condensates for weak and strong homotypic interactions. DNA molecules are represented in pink color and protein molecules are represented in cyan color. In both panels, error bars represent standard errors obtained from block averaging and the associated error bars are smaller than the symbol size.

For evaluating the organization of proteins within the condensate, we calculated the fraction of proteins bound to DNA using a distance-based criterion, where a protein within a distance of σ_P_ + σ_D_ is considered bound to DNA. Figure 3B shows the dependence of this bound fraction on λ_PD_ for different λ_PP_ values. With increasing heterotypic interaction strength one would expect that more proteins will bind to DNA, but this is only true for the case when DNAs are acting as a scaffold for proteins (λ_PP_ = 2.0) (Figure 3B). Whereas with increasing homotypic interactions (λ_PP_ = 3.0, λ_PP_ = 4.0), as proteins form condensates, the trend shows nonmonotonic behavior with increasing heterotypic interaction strength: the fraction of bound proteins initially increases with λ_PD_, peaks at intermediate values (λ_PD_ ≤ 1.0) and then decreases and saturates at higher heterotypic interaction strengths. This nonmonotonic behavior can be explained by considering the effect of DNA compaction. As λ_PD_ increases, DNA becomes more compact (as seen in Figure 3A), which reduces the surface area available for proteins to bind (see snapshots in the inset of Figure 3B). At the same time, proteins are already forming condensates due to strong homotypic interactions, making it harder for them to access tightly packed DNA.

Taken together, both the organization of DNA and the extent of protein–DNA binding within condensates are jointly controlled by homotypic and heterotypic interaction strengths. Even when heterotypic interactions are strong, DNA can become less accessible due to compaction or competition from protein–protein homotypic interactions. These results highlight the complex interplay between protein and DNA interactions in determining the structure and function of biomolecular condensates.

### Role of DNA Length in the Organization of Protein–DNA Condensates

Next, we turn to investigate how the physical properties of DNA affect the structural organization and internal architecture of protein–DNA biomolecular condensates. For this, we first explored the role of DNA chain length in modulating the condensate organization and how the heterotypic and homotypic interactions act upon DNA molecules of varying lengths. Previous experimental studies have shown that longer DNA molecules facilitate protein phase separation by reducing the saturation concentration of proteins for phase separation and forming morphologically different structures of condensates.^28^ Additionally, Ryu et al.^29^ have shown that condensates formed by bridging-induced phase separation have a critical dependency on DNA length. Despite these insights, the different assemblies of a condensate generated due to DNA length remain less explored. To address this, we systematically varied the DNA length across a wide range of 10, 50, 100, 250, 500, and 1000 base pairs while maintaining a constant heterotypic interaction strength at λ_PD_ = 2.0 and explored the influence of three different homotypic interaction strengths of λ_PP_ = 2.0, 3.0, 4.0. This setup allows us to assess how DNA length, in concert with molecular interactions, governs condensate organization.

To quantify DNA organization within the condensate, we computed the average *R*_g_ of DNA chains and normalized it by the *R*_g_ of the same DNA chains in the absence of any attractive interactions, i.e., when both λ_PP_ and λ_PD_ are zero. This *R*_g_ ratio serves as a measure of DNA compaction induced by attractive protein interactions and the result is shown in Figure 4A as a function of DNA length. We observe that for shorter DNA lengths (10 and 50 bps), where persistence length is less than the DNA contour length, these molecules act as rigid rods and undergo negligible compaction for all λ_PP_ values. In contrast, longer DNA molecules (250–1000 bps) show significant compaction, with the degree of compaction depending on the strength of protein–protein interactions. An intriguing trend emerges at an intermediate DNA length of 100 bp, where we observe a pronounced peak in the *R*_g_ ratio for λ_PP_ = 3.0 and 4.0. This suggests that rather than compacting, DNA chains at this length adopt a more expanded and possibly ordered conformation within the condensate (see corresponding snapshot of DNA structures in the inset of Figure 4A). Such behavior may reflect a structural rearrangement wherein proteins form dense networks around the DNA, leading to steric expansion instead of compaction. Interestingly, with increasing DNA lengths, we find that the strongest DNA compaction generally occurs at the lowest homotypic interaction strength (λ_PP_ = 2.0), followed by moderate compaction at λ_PP_ = 3.0, and least compaction at λ_PP_ = 4.0. This inverse relationship between homotypic interaction strength and DNA compaction at fixed heterotypic strength likely arises because strong protein–protein interactions favor self-association of proteins, reducing their availability to engage and wrap around the DNA, thereby attenuating compaction. To evaluate the robustness of these observations, we performed similar analyses under both moderate (λ_PD_ = 0.5) and much stronger (λ_PD_ = 3.5) heterotypic interaction regimes (Figures S4A and S4C). At high λ_PD_ = 3.5, the overall trend of stronger compaction at lower λ_PP_ is preserved, but notably, the peak at 100 bp DNA seen for λ_PP_ = 3.0 and 4.0 vanishes. This indicates that strong protein–DNA interactions lead to uniform compaction across intermediate chain lengths (Figure S4C). Conversely, under moderate heterotypic interaction strength (λ_PD_ = 0.5), we observe that DNA compaction is minimal for λ_PP_ = 2.0 (ratio remains nearly invariant across lengths), while for λ_PP_ = 3.0 and 4.0, compaction increases modestly beyond 100 bp (Figure S4A). For the case of λ_PP_ = 3.0, the result shows relatively higher compaction of DNA than λ_PP_ = 4.0, possibly reflecting an optimal balance between protein–protein association and DNA engagement in this intermediate interaction regime.

**Figure 4:**
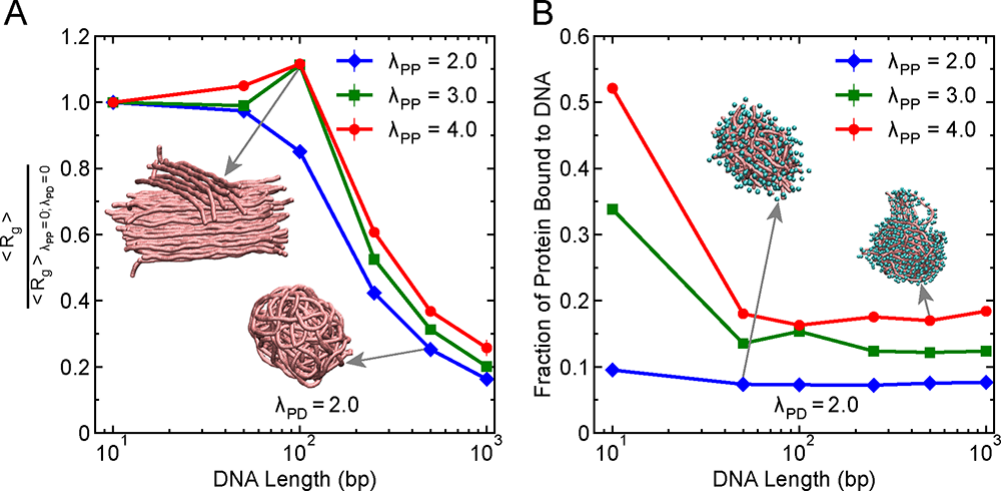
DNA length affects the organization within the condensates. (A) Normalized *R*_g_ of DNA within the condensate, plotted as a function of DNA length for three different homotypic interaction strengths (λ_PP_ = 2.0, 3.0, 4.0) at a fixed heterotypic interaction strength λ_PD_ = 2.0. *R*_g_ is normalized by the *R*_g_ of corresponding DNA chains in the absence of attractive interactions (λ_PP_ = λ_PD_ = 0). Insets show representative conformations of DNA chains (proteins are removed for clarity) at selected lengths and homotypic strengths. (B) Fraction of proteins bound to DNA within the condensate as a function of DNA length, for the same interaction strengths as in panel (A). Insets show the structural organization of proteins bound with DNA at selected lengths and homotypic strengths. DNA molecules are represented in pink color and protein molecules are represented in cyan color. In both panels, error bars represent standard errors obtained from block averaging and the associated error bars are smaller than the symbol size.

To further elucidate the effect of DNA length on protein organization within the condensate, we computed the fraction of proteins bound to DNA as a function of DNA length (Figure 4B). Surprisingly, the fraction of bound proteins remains nearly constant across DNA lengths, except for the shortest chain length (10 bp), which exhibits the highest protein binding. This elevated binding at 10 bp is likely due to the extended, rod-like conformation of these short chains because this DNA length (approximate contour length of 50 Å) is similar to the diameter of the protein, making their surfaces maximally accessible to proteins. For longer chains, despite their increased contour length and compaction, the overall protein–DNA contact fraction remains invariant. This suggests that once a minimal DNA scaffold is available for bridging, further increases in chain length do not significantly alter the bound protein distribution in the condensate. However, increasing the homotypic interaction strength (λ_PP_) consistently leads to higher DNA-bound protein fractions for all DNA lengths (Figure 4B). This trend can be explained by the partial expansion of DNA within the condensate at higher λ_PP_ (as seen in Figure 4A), which increases its surface exposure and accessibility of DNA (see representative snapshots in the inset), thereby facilitating additional protein binding. The same qualitative trends are reproduced in simulations with moderate and strong heterotypic strengths (Figure S4B,D).

### Role of DNA Flexibility in the Organization of Protein–DNA Condensates

Next, we further explored the influence of another critical physical parameter, the flexibility of DNA, on the organization of protein–DNA condensates. DNA flexibility is known to differ significantly between single-stranded DNA (ssDNA) and double-stranded DNA (dsDNA), with ssDNA exhibiting a lower persistence length and higher conformational flexibility. Motivated by this difference, we systematically varied the DNA flexibility of 250 bp long DNA chains by tuning the angular spring constant *K*_θ_ in the bending potential (eq 3), which effectively modulates the DNA’s persistence length (Figure 1B). Specifically, we considered four different *K*_θ_ values: 0.5, 1.5, 7.5, and 20 kcal/mol/rad^2^, chosen such that 0.5 kcal/mol/rad^2^ models the ssDNA flexibility, 1.5 kcal/mol/rad^2^ represents a semiflexible DNA, 7.5 kcal/mol/rad^2^ resembles a semirigid DNA and 20 kcal/mol/rad^2^ captures the dsDNA flexibility (Figure 1C). Recent experiments suggest that flexible DNA increases the propensity for the formation of protein–DNA condensates as the penalty of bending a flexible chain is lower.^30,63,64^ To evaluate how DNA flexibility modulates condensate organization, we computed the average *R*_g_ of DNA and the fraction of proteins bound to DNA within the dense phase for different *K*_θ_ values at a fixed heterotypic interaction strength λ_PD_ = 2.0 for three different homotypic interaction strengths of λ_PP_ = 2.0, 3.0, 4.0.

As shown in Figure 5A, the average DNA *R*_g_ increases monotonically with increasing *K*_θ_, suggesting that more rigid DNA chains maintain a more expanded conformation regardless of homotypic interaction strength. This trend is consistent with expectations from bending rigidity: more rigid chains resist compaction due to the higher energetic cost of deformation. Interestingly, the degree of compaction exhibits a nontrivial dependence on homotypic interaction strength. The most compact conformations are observed at the lowest λ_PP_ = 2.0, followed by moderate compaction at λ_PP_ = 3.0, and the least compaction at λ_PP_ = 4.0, mimicking the similar trend observed in the case of DNA chain length. To test the generality of these results, we performed similar analysis under both moderate (λ_PD_ = 0.5) and much stronger (λ_PD_ = 3.5) heterotypic interaction regimes (Figure S5A,C). At strong λ_PD_ = 3.5, the overall trend of DNA expansion with increasing *K*_θ_ and stronger compaction at lower λ_PP_ is preserved. In contrast, under moderate heterotypic interactions (λ_PD_ = 0.5), DNA compaction is generally minimal, particularly at higher *K*_θ_ values (very high *R*_g_ values, comparable to the *R*_g_ of a single DNA chain in the absence of proteins). Moreover, the ordering of compaction among the different λ_PP_ values shifts, with λ_PP_ = 3.0 showing more compaction than λ_PP_ = 4.0, consistent with trends observed for DNA chain length.

**Figure 5:**
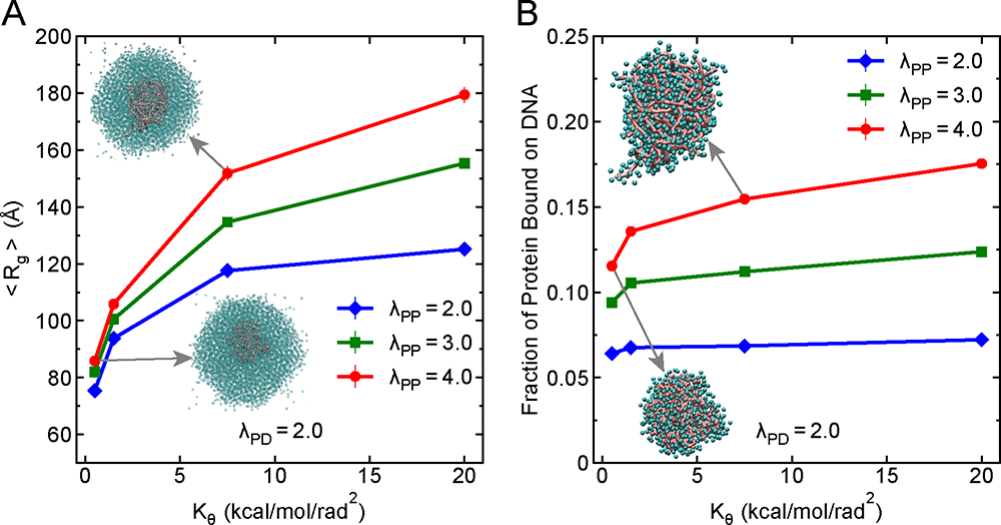
DNA flexibility affects the organization of proteins and DNA within the condensates. (A) Average *R*_g_ of DNA as a function of bending rigidity (*K*_θ_) at a fixed heterotypic interaction strength λ_PD_ = 2.0 for three different homotypic interaction strengths λ_PP_ = 2.0, 3.0, and 4.0. Insets show representative structural organization of DNA chains within the protein–DNA condensate at two selected rigidity values. (B) Fraction of proteins bound to DNA within the dense phase as a function of *K*_θ_ for the same parameter sets as in panel A. Insets show the structural organization of proteins bound with DNA at two selected rigidity values. DNA molecules are represented in pink color and protein molecules are represented in cyan color. Simulations used 250 bp long DNA chains (20 in total) and 5000 protein beads. In both panels, error bars represent standard errors obtained from block averaging and the associated error bars are smaller than the symbol size.

While comparing the protein bound to DNA for different DNA chain flexibility (Figure 5B), we observed that the protein fraction bound to DNA stays constant when the DNA acts as a scaffold to recruit protein and forms condensate (λ_PP_ = 2.0). This observation can be related to the monolayer adsorption of FUS protein on ssDNA and dsDNA, where protein binding to DNA chain under lower protein concentration remains the same independent of flexibility, which is proposed as the mechanism for phase separation.^17^ Increasing homotypic interaction strength further leads to higher DNA-bound protein fractions for all *K*_θ_ values (Figure 5B), suggesting that enhanced homotypic interactions facilitate more favorable DNA accessibility within the condensate. The same qualitative trends are reproduced in simulations at moderate as well as strong heterotypic strengths (Figure S5B,D).

### Multiphasic Nature of Protein–DNA Condensates at Higher Heterotypic Interaction Strengths

While the above results provide crucial insight into the role played by the DNA’s physical properties in organizing the condensate, we next explore how the mere presence of DNA influences the overall phase separation behavior of proteins. Recent studies have shown that the propensity for phase separation of proteins increases with incorporation of DNA molecules in condensates as indicated by the decrease in saturation concentrations of proteins.^22,28,33^ Thus, in order to understand how the phase behavior of proteins changes in the presence of DNA, we varied the interaction strength λ_PP_ between proteins and calculated the coexistence concentrations of proteins in the dense and dilute phases (see Supporting Information for details). For this, we performed simulations under two different conditions: one with moderate protein–DNA interaction (λ_PD_ = 0.5) and another with strong protein–DNA interaction (λ_PD_ = 2.0) strength. In both cases, the system contained 5000 protein molecules and 20 DNA chains, each 250 base pairs long. As a reference, we also simulated a system with proteins only (without any DNA), as shown in Figure 1D. The results are shown in Figure 6A. When the protein–DNA interaction is moderate (λ_PD_ = 0.5), the phase diagram looks similar to that of the protein-only system. The dense and dilute phase concentrations converge to form a typical two-phase coexistence envelope (Figure 1D), indicating no major change in phase behavior. However, when the protein–DNA interaction is strong (λ_PD_ = 2.0), we observe a striking difference: the phase envelope does not converge (see also Figure S6 for other DNA lengths). This nonconverging behavior suggests that the nature of the condensate is fundamentally different under strong heterotypic interactions.

**Figure 6:**
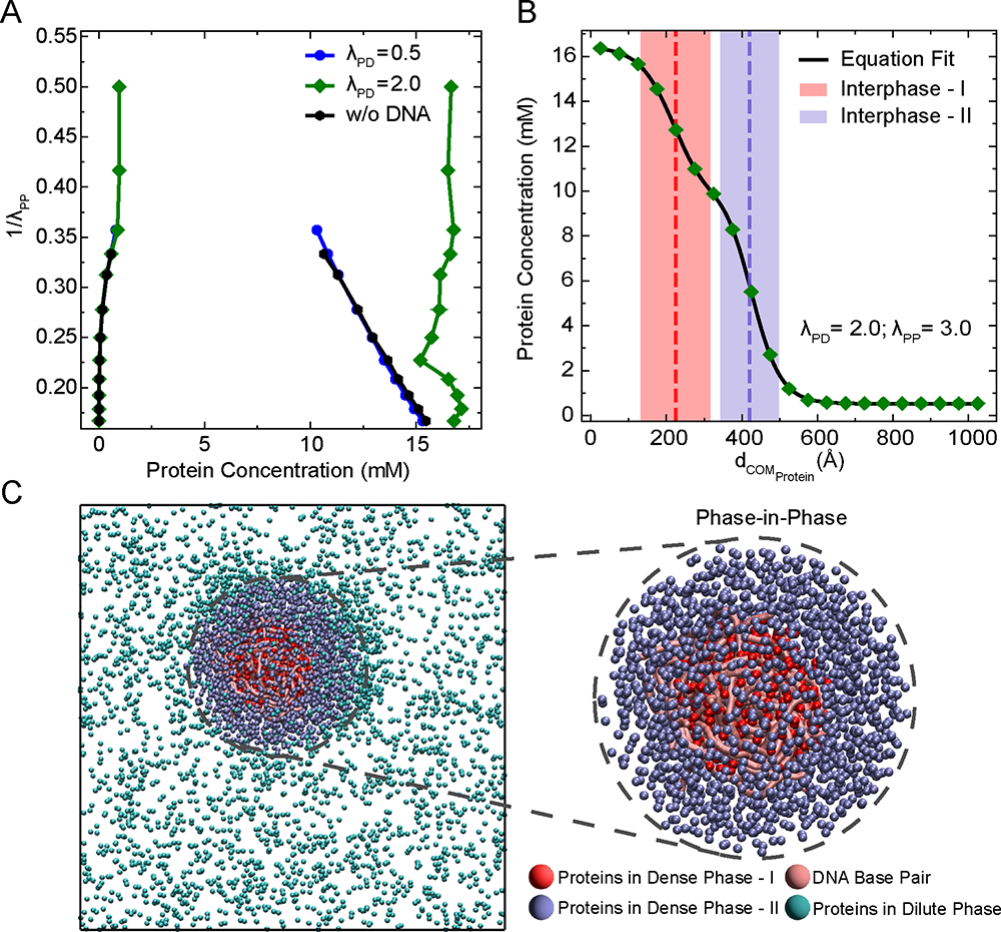
Multiphasic nature of protein–DNA condensates. (A) Phase diagrams showing protein concentrations in the dilute and dense phases as a function of protein–protein interaction strength (λ_PP_), for three systems: proteins only (black), proteins with DNA at moderate (λ_PD_ = 0.5, blue) and strong (λ_PD_ = 2.0, green) heterotypic interaction strength. (B) Radial density profile of proteins from the center of mass (COM) of proteins in the largest cluster at λ_PD_ = 2.0 and λ_PP_ = 3.0, showing two distinct protein populations. The profile is fitted with a double hyperbolic tangent function (solid black line), capturing the presence of an inner DNA-associated protein-rich region (dense phase-I) and an outer protein-rich region not in contact with DNA (dense phase-II). Red and blue dotted lines represent the location of the two interphases between dense phase-I, dense phase-II and between dense phase-II and dilute phase, respectively. (C) Representative simulation snapshot corresponding to panel B, illustrating the multiphasic organization. DNA-bound proteins (dense phase-I) are shown in red, unbound proteins (dense phase-II) in blue, DNA molecules in pink, and proteins in the dilute phase in cyan.

To understand this difference more clearly, we analyzed the spatial distribution of proteins within the condensate at λ_PD_ = 2.0 and λ_PP_ = 3.0, a condition corresponding to the nonconverging regime (see also Figure S7 for other combinations of λ_PD_ and λ_PP_). Specifically, we looked at how the protein density changes with distance from the center of mass (COM) of proteins in the largest cluster (see Supporting Information). As shown in Figure 6B, the radial density profile reveals two distinct regions of proteins within the condensate, which can be clearly captured by fitting a double hyperbolic tangent function to the radial density distribution (see Supporting Information for more details). These two regions represent two types of protein populations within the same condensate. The first population consists of proteins that are bound to DNA and clustered around it, forming the inner phase (Dense phase-I) differentiated within the condensate by the interphase-I, shown as the red-shaded region in Figure 6B. The second population consists of proteins that are not bound to DNA and are distributed away from the DNA, forming the outer phase (Dense phase-II) distinguished from the dilute phase by the interphase-II, shown as the blue-shaded region. A simulation snapshot in Figure 6C further illustrates this structure, where red-colored proteins are DNA-bound (Dense phase-I), and blue-colored proteins are unbound (Dense phase-II). This observation indicates that the condensate is not uniform in composition, but rather contains multiple subphases, a phenomenon usually referred to as “multiphasic organization”.^65–67^ In other words, even though all proteins are chemically identical, their interactions with DNA create a spatial separation within the condensate, leading to a “phase-in-phase” architecture where different regions have different protein densities. Such architectures of multiphasic condensate have been reported in the nucleolus, that has distinct immiscible phases^5,68^ and for the case of a ternary mixture of protein HP1, histone protein H1, and DNA where a layered organization was formed in the condensate which may help with DNA packaging within the condensate.^33^

To further probe the requirements for the emergence of phase-in-phase organization, we examined the protein density profiles and the gradient of protein concentration as a function of distance from the protein center of mass in condensates formed with DNA chains of varying lengths (10, 50, 100, 500 bp) at λ_PD_ = 2.0 and λ_PP_ = 3.0 (Figure S8). We find that very short DNA (∼10 bp) does not exhibit the phase-in-phase morphology observed in Figure 6B, suggesting that a minimal contour length is necessary to nucleate and stabilize such spatial segregation. For longer DNA chain lengths (50–500 bp), phase-in-phase architecture persists. To test whether the DNA chain length threshold persists at higher λ_PD_, we performed a similar analysis for λ_PD_ = 2.5, 3.0, and 3.5 (Figure S9). We find that stronger heterotypic interactions override the DNA chain length requirement, enabling even 10 bp DNA chains to exhibit phase-in-phase assembly. We next varied DNA flexibility (*K*_θ_ = 0.5, 1.5, 7.5, 20 kcal/mol/rad^2^) at a fixed chain length of 250 bp (Figure S10) and found that phase-in-phase organization occurs for all stiffness values considered. Together, these results indicate that phase-in-phase organization depends more strongly on heterotypic interaction strength than on DNA chain length or flexibility.

### Differential Protein Dynamics within the Protein–DNA Condensate

As we observed the formation of distinct protein phases within a protein–DNA condensate for strong heterotypic interaction strengths, we hypothesized that these distinct protein phases might exhibit different dynamic behaviors. To evaluate the dynamics of proteins for multiple phases, the use of mean square displacement (MSD) of proteins for the calculation of the diffusion coefficient (*D*) poses challenges, as it would average all the distinct modes of diffusion. To overcome this limitation, we employed an alternative method to compute *D* using the probability distribution of displacements, *P*(*r*, *t*), in the radial direction (*r*) as a function of lag time *t*.^69^ This method captures the presence of multiple diffusion behaviors by fitting the displacement distribution to a sum of Gaussian components. Each component is characterized by a distinct diffusion coefficient (*D*_*i*_) and its corresponding fractional population (*p*_*i*_), described by the expression:

$$P(r,t)=\sum_{i=1}^{n}p_{i}\frac{4\pi r^{2}}{(4\pi D_{i}t{)}^{3/2}}e^{-r^{2}/4D_{i}t},\;\mathrm{with}\sum_{i=1}^{n}p_{i}=1$$ 6

where *n* is the number of diffusivity (*D*) values that we increase for fitting the probability distribution until the fit achieves minimal residuals.

We find that it is necessary to include multiple diffusivity (*D*) values to fit the displacement distribution data, which indicates that there are different modes of diffusion for the proteins. For instance, we first applied this method to a system with weak heterotypic interaction strength (λ_PD_ = 0.3) and strong homotypic interaction (λ_PP_ = 3.0), under which DNA is not recruited in the condensate (see also Figure 2). In Figure S11A, the corresponding probability distribution is shown for different lag time *t* and we found that we need three *D* values (*n* = 3) to best fit the displacement probability distribution, suggesting three separate modes of diffusion. The quality of the fit is confirmed by the residual, which is the difference between fitted values and the simulation data points, and shows the minimal deviation across *r* (lower panel in Figure S11A). We then checked these three *D* values obtained from the fitting, which remained constant across lag times, as shown in Figure S11B, and likely correspond to (i) fast-diffusing proteins in the dilute phase (*D*_1_), (ii) proteins at the interface between dense and dilute phases (*D*_2_), and (iii) slower-diffusing proteins deeply embedded in the condensed phase (*D*_3_).

Next, we analyzed this behavior for the multiphasic protein condensate formed under strong heterotypic (λ_PD_ = 2.0) and homotypic (λ_PP_ = 3.0) interaction strengths. Remarkably, in this case, we found that five distinct diffusivity modes (*n* = 5) were required for an optimal fit to accurately describe the displacement distributions, as any fewer Gaussian components failed to capture the peaks and troughs of the distribution, and adding an additional mode of diffusion (*n* = 6) did not significantly improve the residuals or the fit (Figures 7A, S12, and S13), revealing a more complex dynamical landscape. These diffusivity components remained constant across lag times (Figure 7B), indicating that each mode exhibits stable diffusivity across lag times and their averaged values are shown in Figure 7C. Comparison with the DNA-excluded condensate clearly reveals two additional slow modes of diffusion (*D*_4_ and *D*_5_) in the DNA-recruited system (Figure 7C). Based on the ordering of the diffusion coefficients (*D*_5_ < *D*_4_ < *D*_3_ < *D*_2_ < *D*_1_), we propose a possible interpretation of these five dynamic populations. The slowest diffusion component (*D*_5_) likely corresponds to proteins in the dense phase that are either immobilized by forming bridging contacts with DNA or are strongly bound to DNA. The next two modes (*D*_4_ and *D*_3_) may represent proteins bound on the DNA surface and those diffusing within the condensed phase but not in contact with DNA. The fourth diffusion mode (*D*_2_) corresponds to proteins at the interface of condensed and dilute phase and the fastest mode (*D*_1_) represents proteins in the dilute phase. To assess the sensitivity of these results to fitting parameters, we repeated the analysis with four different bin sizes (300, 500, 1000, and 2000) for the displacement distributions. In all cases, the five diffusion modes persisted with similar ordering, and the fitted diffusion coefficients were unaffected by the choice of bin size (Figure S14). These results thus emphasize that even within a spatially continuous condensate, proteins experience diverse microenvironments due to differential DNA interactions. Notably, such differential nature of protein dynamics has been experimentally observed in HP1α chromatin condensate where the proteins diffusivity varies with their distance from the chromatin in the condensate.^70^ Experimentally, fluorescence recovery after photobleaching (FRAP) is the primary method used for determining protein dynamics in condensates where a region of fluorescent proteins is photobleached and the time required for the recovery of fluorescence in this region is interpreted as a measure of protein dynamics.^71,72^ Our findings on differential protein dynamics within a condensate underscore the importance of selecting the appropriate region for studying the protein dynamics within a condensate, as different regions of proteins within condensate reflect different dynamic properties of proteins due to recruitment of DNA.

**Figure 7:**
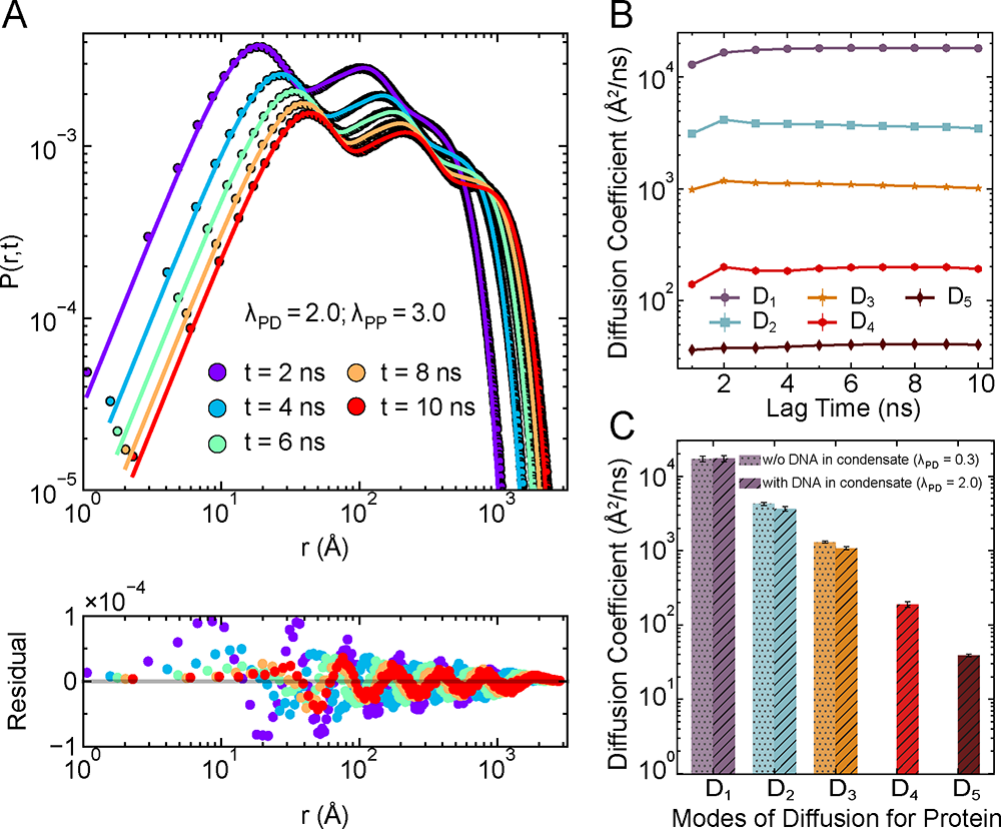
Differential dynamics of protein within protein–DNA condensates. (A) Radial displacement probability distributions of proteins, *P*(*r*, *t*), for different lag times *t*, under strong heterotypic (λ_PD_ = 2.0) and homotypic (λ_PP_ = 3.0) interaction strengths. The points denote histogram data from simulations, while solid lines show the best-fit using a sum of five Gaussian components (eq 6). The difference between the Gaussian fitted lines and the simulation data points are represented as residuals from the data fitting for each lag time *t* (lower panel), confirming the quality of fit (see also Figures S12 and S13). (B) Diffusion coefficients (*D*_*i*_) corresponding to the five dynamic modes extracted from the Gaussian fits in (A), shown as a function of lag time. The plateau behavior indicates that each mode exhibits stable diffusivity across lag times. (C) Comparison of the average diffusion coefficients for different dynamic modes between the DNA-recruited condensate (λ_PD_ = 2.0, hatched bars) and DNA-excluded condensate (λ_PD_ = 0.3, dotted bars). The DNA-recruited case reveals two additional slow diffusion modes (*D*_4_ and *D*_5_), highlighting the emergence of new dynamic populations due to DNA localization within the condensate.

## Conclusions

In this study, we employed a minimalist coarse-grained model to investigate how heterotypic protein–DNA and homotypic protein–protein interactions, along with DNA physical properties such as chain length and flexibility, influence the formation, structure, and dynamics of protein–DNA condensates. Our simulations revealed that while homotypic interactions are sufficient to drive protein condensation, heterotypic interactions with DNA greatly influence the phase behavior and condensate formation. We found that DNA acts as a scaffold for recruiting proteins when heterotypic interactions are stronger, leading to the compaction of DNA and formation of condensates. We further demonstrated that longer DNA chains and more flexible DNA molecules undergo a higher degree of compaction and promote the formation of distinct structural morphologies within condensates, including multiphasic organizations. These structural features are regulated by the strength of homotypic and heterotypic interactions.

Importantly, our results highlighted the existence of distinct protein microenvironments within the condensate, with protein populations exhibiting different binding modes, ranging from strongly DNA-bound to completely unbound and freely diffusing in the dilute phase. These diverse structural states lead to markedly different diffusion behaviors. By analyzing the distributions of radial displacements of proteins, we uncovered up to five distinct diffusion modes, with the slowest corresponding to strongly DNA-bound proteins and the fastest to freely diffusing ones. Such findings resonate with experimental observations of spatially dependent protein dynamics in chromatin-associated condensates and provide a physical basis for interpreting heterogeneous fluorescence recovery signals seen in FRAP experiments.

Our work demonstrates that DNA is not merely a passive scaffold but an active modulator of condensate architecture and dynamics, capable of recruiting proteins, altering their spatial organization, and modulating their mobility. This mechanistic understanding is crucial for interpreting the functional consequences of biomolecular condensates in vivo, particularly in chromatin organization, transcriptional regulation, and other DNA-templated processes.

While our CG simulation results present a plausible microscopic picture of the formation of protein–DNA condensates, it is important to discuss its limitations. The proteins and DNA are represented using simplified models that lack sequence specificity and do not capture detailed structural features such as helical twisting of DNA or the multidomain architecture of real proteins. Furthermore, the model neglects explicit solvent and electrostatic effects, which may play important roles in determining the phase behavior and internal structuring of real biomolecular condensates. Moreover, our model also does not include specific interactions between proteins or between proteins and DNA such as multivalent domain-mediated contacts, DNA-binding motifs, or charge patterning effects, which are known to significantly influence condensate composition, selectivity, and material properties.^38,73,74^ Nevertheless, despite these simplifications, our model successfully recapitulates key qualitative features observed experimentally and provides a foundational framework for understanding the interplay between molecular properties and phase organization in protein–DNA condensates. Importantly, the same modeling framework can be readily adapted to protein–RNA systems by appropriately parametrizing the RNA polymer properties (e.g., persistence length) and protein–RNA interaction parameters to capture the relevant physical characteristics of the target system. This work lays the groundwork for future studies incorporating more detailed molecular representations and sequence-specific interactions to bridge the gap between minimal models and biological complexity.

## Supplementary Material



## Data Availability

All data are included in the manuscript and/or Supporting Information.

## Notes

Calculation of protein–DNA dissociation constant (*K*_D_); calculation of DNA persistence length; calculation of radius of gyration­(*R*_g_) of DNA chains; analysis of radial density profile of proteins in single and multiphasic condensates; protein–DNA dissociation constant *K*_D_ as a function of their interaction strengths; dilute phase protein concentration as a function of simulation time at λ_PD_ = 0.3, λ_PP_ = 3.0; probability distribution of cluster sizes for λ_PD_ = 2.0 and λ_PP_ = 3.0; normalized *R*_g_ of DNA molecules and fraction of proteins bound to DNA as a function of DNA length for moderate (λ_PD_ = 0.5) and strong (λ_PD_ = 3.5) heterotypic interaction strengths with three different homotypic interaction strengths; average *R*_g_ of DNA of length 250 bp and fraction of proteins bound to DNA as a function of bending rigidity *K*_θ_ for moderate (λ_PD_ = 0.5) and strong (λ_PD_ = 3.5) heterotypic interaction strengths with three different homotypic interaction strengths; phase diagram of protein concentrations in the dilute and dense phases as a function of λ_PP_ for three different DNA lengths at strong λ_PD_ = 2.0; radial density profile of proteins from the center of mass of proteins in the largest cluster at λ_PD_ = 2.5, λ_PP_ = 3.6 and λ_PD_ = 2.5, λ_PP_ = 4.0; radial density profile of proteins at λ_PD_ = 2.0, λ_PP_ = 3.0 for different DNA length; radial density profile of proteins in condensate formed with 10 bp DNA at λ_PP_ = 3.0 for different λ_PD_ ;radial density profile of proteins at λ_PD_ = 2.0, λ_PP_ = 3.0 for different bending rigidity *K*_θ_; displacement distributions of proteins for different lag times at λ_PD_ = 0.3, λ_PP_ = 3.0 and diffusion coefficients corresponding to the three dynamic modes of proteins as a function of lag time; residual analysis for Gaussian components of protein displacement distribution in multiphase condensates; comparison of Gaussian fits with different number of components for protein displacement distribution in multiphase condensates; and comparison of the average diffusion coefficients for different bin sizes in the displacement distribution (PDF)

## References

[1] Shin Y., Brangwynne C. P. (2017). Liquid phase condensation in cell physiology and disease. Science.

[2] Banani S. F., Lee H. O., Hyman A. A., Rosen M. K. (2017). Biomolecular condensates:
organizers of cellular biochemistry. Nat. Rev.
Mol. Cell Biol..

[3] Mao Y. S., Zhang B., Spector D. L. (2011). Biogenesis and function of nuclear
bodies. Trends in Genetics.

[4] Decker C. J., Parker R. (2012). P-bodies and stress
granules: possible roles in the
control of translation and mRNA degradation. Cold Spring Harbor perspectives in biology.

[5] Feric M., Vaidya N., Harmon T. S., Mitrea D. M., Zhu L., Richardson T. M., Kriwacki R. W., Pappu R. V., Brangwynne C. P. (2016). Coexisting
liquid phases underlie nucleolar subcompartments. Cell.

[6] Feric M., Demarest T. G., Tian J., Croteau D. L., Bohr V. A., Misteli T. (2021). Self-assembly of multi-component mitochondrial nucleoids
via phase separation. EMBO J..

[7] Hyman A. A., Weber C. A., Jülicher F. (2014). Liquid-liquid
phase separation in
biology. Annual review of cell and developmental
biology.

[8] Li P., Banjade S., Cheng H.-C., Kim S., Chen B., Guo L., Llaguno M., Hollingsworth J. V., King D. S., Banani S. F. (2012). Phase transitions in the assembly of multivalent signalling proteins. Nature.

[9] King J. T., Shakya A. (2021). Phase separation of DNA: From past to present. Biophysical journal.

[10] Dignon G. L., Best R. B., Mittal J. (2020). Biomolecular phase
separation: from
molecular driving forces to macroscopic properties. Annu. Rev. Phys. Chem..

[11] Murthy A. C., Tang W. S., Jovic N., Janke A. M., Seo D. H., Perdikari T. M., Mittal J., Fawzi N. L. (2021). Molecular interactions
contributing to FUS SYGQ LC-RGG phase separation and co-partitioning
with RNA polymerase II heptads. Nature structural
& molecular biology.

[12] Mondal A., Bhattacherjee A. (2015). Searching target sites on DNA by
proteins: Role of
DNA dynamics under confinement. Nucleic Acids
Res..

[13] Mohanty P., Kapoor U., Sundaravadivelu
Devarajan D., Phan T. M., Rizuan A., Mittal J. (2022). Principles
governing the phase separation
of multidomain proteins. Biochemistry.

[14] Rekhi S., Garcia C. G., Barai M., Rizuan A., Schuster B. S., Kiick K. L., Mittal J. (2024). Expanding
the molecular language
of protein liquid–liquid phase separation. Nature Chem..

[15] Larson A. G., Elnatan D., Keenen M. M., Trnka M. J., Johnston J. B., Burlingame A. L., Agard D. A., Redding S., Narlikar G. J. (2017). Liquid
droplet formation by HP1 suggests a role for phase separation in heterochromatin. Nature.

[16] Laghmach R., Di Pierro M., Potoyan D. A. (2021). The interplay of chromatin phase
separation and lamina interactions in nuclear organization. Biophys. J..

[17] Renger R., Morin J. A., Lemaitre R., Ruer-Gruss M., Jülicher F., Hermann A., Grill S. W. (2022). Co-condensation
of proteins with single- and double-stranded DNA. Proc. Natl. Acad. Sci. U. S. A..

[18] Shrinivas K., Sabari B. R., Coffey E. L., Klein I. A., Boija A., Zamudio A. V., Schuijers J., Hannett N. M., Sharp P. A., Young R. A. (2019). Enhancer features that drive formation of transcriptional
condensates. Molecular cell.

[19] Qamar S., Wang G., Randle S. J., Ruggeri F. S., Varela J. A., Lin J. Q., Phillips E. C., Miyashita A., Williams D., Ströhl F. (2018). FUS phase separation
is modulated by a molecular chaperone and methylation of arginine
cation-π interactions. Cell.

[20] Gibson B. A., Doolittle L. K., Schneider M. W., Jensen L. E., Gamarra N., Henry L., Gerlich D. W., Redding S., Rosen M. K. (2019). Organization
of chromatin by intrinsic and regulated phase separation. Cell.

[21] Brackley C. A., Liebchen B., Michieletto D., Mouvet F., Cook P. R., Marenduzzo D. (2017). Ephemeral protein binding to DNA shapes stable nuclear
bodies and chromatin domains. Biophysical journal.

[22] Phan T. M., Kim Y. C., Debelouchina G. T., Mittal J. (2024). Interplay between charge
distribution and DNA in shaping HP1 paralog phase separation and localization. eLife.

[23] Her C., Phan T. M., Jovic N., Kapoor U., Ackermann B. E., Rizuan A., Kim Y. C., Mittal J., Debelouchina G. T. (2022). Molecular
interactions underlying the phase separation of HP1: role of phosphorylation,
ligand and nucleic acid binding. Nucleic Acids
Res..

[24] Quail T., Golfier S., Elsner M., Ishihara K., Murugesan V., Renger R., Jülicher F., Brugués J. (2021). Force generation
by protein-DNA co-condensation. Nat. Phys..

[25] Haugk L., Merlitz H., Sommer J.-U. (2024). Single
chain polymer condensates
under external force. Macromolecules.

[26] Monahan Z., Ryan V. H., Janke A. M., Burke K. A., Rhoads S. N., Zerze G. H., O’Meally R., Dignon G. L., Conicella A. E., Zheng W. (2017). Phosphorylation
of the FUS low-complexity domain disrupts
phase separation, aggregation, and toxicity. EMBO journal.

[27] Hazra M. K., Levy Y. (2023). Cross-talk of cation-π interactions with electrostatic and
aromatic interactions: A salt-dependent trade-off in biomolecular
condensates. journal of physical chemistry letters.

[28] Keenen M. M., Brown D., Brennan L. D., Renger R., Khoo H., Carlson C. R., Huang B., Grill S. W., Narlikar G. J., Redding S. (2021). HP1 proteins compact
DNA into mechanically and positionally
stable phase separated domains. eLife.

[29] Ryu J.-K., Bouchoux C., Liu H. W., Kim E., Minamino M., de Groot R., Katan A. J., Bonato A., Marenduzzo D., Michieletto D. (2021). Bridging-induced phase
separation induced by
cohesin SMC protein complexes. Sci. Adv..

[30] Shakya A., King J. T. (2018). DNA local-flexibility-dependent assembly
of phase-separated
liquid droplets. Biophysical journal.

[31] Kapoor U., Kim Y. C., Mittal J. (2024). Coarse-grained
models to study protein–DNA
interactions and liquid–liquid phase separation. J. Chem. Theory Comput..

[32] Yasuda I., von Bülow S., Tesei G., Yamamoto E., Yasuoka K., Lindorff-Larsen K. (2025). Coarse-grained model of disordered
RNA for simulations
of biomolecular condensates. J. Chem. Theory
Comput..

[33] Latham A. P., Zhang B. (2022). On the stability and layered organization of protein-DNA condensates. Biophys. J..

[34] Ancona M., Brackley C. A. (2022). Simulating the chromatin-mediated
phase separation
of model proteins with multiple domains. Biophys.
J..

[35] Schede H. H., Natarajan P., Chakraborty A. K., Shrinivas K. (2023). A model for
organization and regulation of nuclear condensates by gene activity. Nat. Commun..

[36] Snead W. T., Skillicorn M. K., Shrinivas K., Gladfelter A. S. (2025). Immiscible
proteins compete for RNA binding to order condensate layers. Proc. Natl. Acad. Sci. U. S. A..

[37] Dignon G. L., Zheng W., Kim Y. C., Best R. B., Mittal J. (2018). Sequence determinants
of protein phase behavior from a coarse-grained model. PLoS computational biology.

[38] Sundaravadivelu
Devarajan D., Wang J., Szała-Mendyk B., Rekhi S., Nikoubashman A., Kim Y. C., Mittal J. (1912). Sequence-dependent
material properties of biomolecular condensates and their relation
to dilute phase conformations. Nat. Commun..

[39] Miller C. M., Kim Y. C., Mittal J. (2016). Protein composition
determines the
effect of crowding on the properties of disordered proteins. Biophysical journal.

[40] Welles R. M., Sojitra K. A., Garabedian M. V., Xia B., Wang W., Guan M., Regy R. M., Gallagher E. R., Hammer D. A., Mittal J. (2024). Determinants that enable
disordered protein assembly into discrete condensed phases. Nat. Chem..

[41] Statt A., Casademunt H., Brangwynne C. P., Panagiotopoulos A. Z. (2020). Model for
disordered proteins with strongly sequence-dependent liquid phase
behavior. J. Chem. Phys..

[42] Rana U., Brangwynne C. P., Panagiotopoulos A. Z. (2021). Phase separation vs aggregation behavior
for model disordered proteins. J. Chem. Phys..

[43] Brackley C. A., Taylor S., Papantonis A., Cook P. R., Marenduzzo D. (2013). Nonspecific
bridging-induced attraction drives clustering of DNA-binding proteins
and genome organization. Proc. Natl. Acad. Sci.
U. S. A..

[44] Rubio-Cosials A., Sydow J. F., Jiménez-Menéndez N., Fernández-Millán P., Montoya J., Jacobs H. T., Coll M., Bernadó P., Solà M. (2011). Human mitochondrial
transcription factor A induces a U-turn structure in the light strand
promoter. Nature structural & molecular
biology.

[45] Manning G.
S. (2006). The persistence
length of DNA is reached from the persistence length of its null isomer
through an internal electrostatic stretching force. Biophysical journal.

[46] Mitchell J. S., Glowacki J., Grandchamp A. E., Manning R. S., Maddocks J. H. (2017). Sequence-dependent
persistence lengths of DNA. J. Chem. Theory
Comput..

[47] Ashbaugh H. S., Hatch H. W. (2008). Natively unfolded protein stability as a coil-to-globule
transition in charge/hydropathy space. J. Am.
Chem. Soc..

[48] Wang X., Schwartz J. C., Cech T. R. (2015). Nucleic
acid-binding specificity
of human FUS protein. Nucleic acids research.

[49] Anderson J. A., Lorenz C. D., Travesset A. (2008). General purpose
molecular dynamics
simulations fully implemented on graphics processing units. J. Comput. Phys..

[50] Anderson J. A., Glaser J., Glotzer S. C. (2020). HOOMD-blue: A Python
package for
high-performance molecular dynamics and hard particle Monte Carlo
simulations. Comput. Mater. Sci..

[51] Ramasubramani V., Dice B. D., Harper E. S., Spellings M. P., Anderson J. A., Glotzer S. C. (2020). freud: A software
suite for high
throughput analysis of particle simulation data. Comput. Phys. Commun..

[52] Patel A., Lee H. O., Jawerth L., Maharana S., Jahnel M., Hein M. Y., Stoynov S., Mahamid J., Saha S., Franzmann T. M. (2015). A liquid-to-solid phase
transition of the ALS
protein FUS accelerated by disease mutation. Cell.

[53] Murthy A. C., Dignon G. L., Kan Y., Zerze G. H., Parekh S. H., Mittal J., Fawzi N. L. (2019). Molecular interactions underlying
liquid-liquid phase separation of the FUS low-complexity domain. Nature structural & molecular biology.

[54] Nott T. J., Petsalaki E., Farber P., Jervis D., Fussner E., Plochowietz A., Craggs T. D., Bazett-Jones D. P., Pawson T., Forman-Kay J. D. (2015). Phase transition of
a disordered nuage protein generates environmentally responsive membraneless
organelles. Molecular cell.

[55] Stortz M., Presman D. M., Levi V. (2024). Transcriptional
condensates: a blessing
or a curse for gene regulation?. Commun. Biol..

[56] Brackley C. A., Marenduzzo D. (2020). Bridging-induced microphase separation:
photobleaching
experiments, chromatin domains and the need for active reactions. Briefings in functional genomics.

[57] Shahu S., Vtyurina N., Das M., Meyer A. S., Ganji M., Abbondanzieri E. A. (2024). Bridging
DNA contacts allow Dps from E. coli to condense
DNA. Nucleic Acids Res..

[58] Morin J. A., Wittmann S., Choubey S., Klosin A., Golfier S., Hyman A. A., Jülicher F., Grill S. W. (2022). Sequence-dependent
surface condensation of a pioneer transcription factor on DNA. Nat. Phys..

[59] Ahlawat V., Dhiman A., Mudiyanselage H. E., Zhou H.-X. (2024). Protamine-mediated
tangles produce extreme deoxyribonucleic acid compaction. J. Am. Chem. Soc..

[60] Chhetri K. B., Jang Y. H., Lansac Y., Maiti P. K. (2022). Effect of phosphorylation
of protamine-like cationic peptide on the binding affinity to DNA. Biophys. J..

[61] Kim Y. J., Lee M., Lee Y.-T., Jing J., Sanders J. T., Botten G. A., He L., Lyu J., Zhang Y., Mettlen M. (2023). Light-activated macromolecular
phase separation modulates transcription by reconfiguring chromatin
interactions. Sci. Adv..

[62] Swain P., Choubey S., Vemparala S. (2024). Role of protein–protein interactions
on organization and dynamics of a model chromatin. J. Chem. Phys..

[63] André A. A., Spruijt E. (2018). Rigidity rules in DNA
droplets: Nucleic acid flexibility
affects model membraneless organelles. Biophys.
J..

[64] Shakya A., Girard M., King J. T., Olvera de la Cruz M. (2020). Role of chain
flexibility in asymmetric polyelectrolyte complexation in salt solutions. Macromolecules.

[65] Kelley F. M., Favetta B., Regy R. M., Mittal J., Schuster B. S. (2021). Amphiphilic
proteins coassemble into multiphasic condensates and act as biomolecular
surfactants. Proc. Natl. Acad. Sci. U. S. A..

[66] Pandey V., Hosokawa T., Hayashi Y., Urakubo H. (2025). Multiphasic
protein
condensation governed by shape and valency. Cell Reports.

[67] Rana U., Xu K., Narayanan A., Walls M. T., Panagiotopoulos A. Z., Avalos J. L., Brangwynne C. P. (2024). Asymmetric oligomerization state
and sequence patterning can tune multiphase condensate miscibility. Nature Chem..

[68] Lafontaine D. L., Riback J. A., Bascetin R., Brangwynne C. P. (2021). The nucleolus
as a multiphase liquid condensate. Nat. Rev.
Mol. Cell Biol..

[69] Zheng W., Dignon G. L., Jovic N., Xu X., Regy R. M., Fawzi N. L., Kim Y. C., Best R. B., Mittal J. (2020). Molecular
details of protein condensates probed by microsecond long atomistic
simulations. J. Phys. Chem. B.

[70] Strom A. R., Emelyanov A. V., Mir M., Fyodorov D. V., Darzacq X., Karpen G. H. (2017). Phase separation
drives heterochromatin domain formation. Nature.

[71] Taylor N. O., Wei M.-T., Stone H. A., Brangwynne C. P. (2019). Quantifying
dynamics in phase-separated condensates using fluorescence recovery
after photobleaching. Biophysical journal.

[72] Soranno A. (2019). The trap in
the FRAP: a cautionary tale about transport measurements in biomolecular
condensates. Biophysical journal.

[73] Devarajan D. S., Rekhi S., Nikoubashman A., Kim Y. C., Howard M. P., Mittal J. (2022). Effect of charge distribution
on the dynamics of polyampholytic
disordered proteins. Macromolecules.

[74] Maurici N., Phan T. M., Henty-Ridilla J. L., Kim Y. C., Mittal J., Bah A. (2025). Uncovering the molecular
interactions underlying MBD2 and MBD3 phase
separation. J. Phys. Chem. B.

