# Supplementary material for: Multiphasic Organization and Differential Dynamics of Proteins within Protein–DNA Biomolecular Condensates
Source: J Phys Chem B. 2025 Sep 15;129(38):9588–99. doi: 10.1021/acs.jpcb.5c03987 (PMC12478860; doi:10.1021/acs.jpcb.5c03987)
Supplement: Supplementary file 1 [file jp5c03987_si_001.pdf]

Supporting Information: Multiphasic Organization and Differential Dynamics of Proteins Within Protein-DNA Biomolecular Condensates

Ashish Shyam Tangade,[†] Anupam Mondal,[†] Jiahui Wang,[†] Young C. Kim,[‡] and Jeetain Mittal[*,†,¶,§]

†*Artie McFerrin Department of Chemical Engineering, Texas A&M University, College Station, Texas 77843, USA*

‡*Center for Materials Physics and Technology, Naval Research Laboratory, Washington, District of Columbia 20375, USA*

¶*Department of Chemistry, Texas A&M University, College Station, Texas 77843, USA*

§*Interdisciplinary Graduate Program in Genetics and Genomics, Texas A&M University, College Station, Texas 77843, USA*

E-mail: jeetain@tamu.edu

In this supporting information, we present supporting text and additional figures that were referenced in the main text.

Calculation of Protein-DNA Dissociation Constant (K_D)

Protein-DNA dissociation constant (K_D) was calculated from fraction of bound states of a protein on DNA using the formula mentioned by Lopez et al.[1]:

$$K_D = \frac{(1 - p_b)^2}{N_A V p_b} \tag{S1}$$

where p_b is the fraction of bound states; N_A is the Avogadro's constant and V is the volume of simulation box. The fraction of bound states was computed from the potential of mean force (PMF), using the criterion that a bound state is considered when PMF < -1 k_BT. The PMF was calculated using umbrella sampling simulations performed in the LAMMPS simulation software package[2] using COLVARS module.[3] Each simulation was conducted in a cubic box of edge length 200 Å containing a single protein molecule and a single 50 bp DNA chain. For umbrella sampling 27 windows were considered for a distance range of 0 to 81 Å with a harmonic bias potential with a spring constant of 0.2 kcal/mol/Å^2. Each window was simulated for 100 ns using a timestep of 10 fs.

Calculation of DNA Persistence Length

The persistence length (l_p) of DNA chains is calculated using the polymer analysis library from the MDAnalysis[4] python package. This approach relies on evaluating the orientational correlations between bond vectors along the polymer. The autocorrelation function $C(n)$ between bond vectors separated by n bonds is computed as:

$$C(n) = \langle \cos\theta_{i,i+n} \rangle = \langle \mathbf{a_i} \cdot \mathbf{a_{i+n}} \rangle \tag{S2}$$

where $\mathbf{a_i}$ and $\mathbf{a_{i+n}}$ are unit vectors along bonds i and $i + n$, respectively, and the angular bracket denotes an ensemble average over all pairs separated by n bonds.

To extract the persistence length, the computed autocorrelation function is fit to the

theoretical exponential decay:

$$C(n) \approx \exp\left(-\frac{nl_b}{l_p}\right) \tag{S3}$$

where $l_b = 5.5$ Å is the average bond length between adjacent DNA beads. By fitting Eq. (S3) to the data obtained from Eq. (S2), the persistence length l_p of the DNA chain is estimated.

Radius of Gyration Calculations for DNA Molecules

We computed the radius of gyration (R_g) of DNA chains by calculating the gyration tensor as[5] :

$$\mathbf{G} = \frac{1}{M}\sum_{i=1}^{N} m_i\,\Delta\mathbf{r}_i\,\Delta\mathbf{r}_i \tag{S4}$$

where $\Delta\mathbf{r}_i$ is the vector to i^{th} monomer from the chain's center of mass, m_i is the mass of the ith monomer, M is the total mass of the chain and N is the total number of monomer. From this gyration tensor, we calculate the radius of gyration of the DNA chains as the square root of the trace of the gyration tensor $R_g = \langle \mathrm{tr}\,\mathbf{G}\rangle^{1/2}$.

Radial Density Profile Analysis of Proteins in Single and Multiphasic Condensates

For calculating the density profile of proteins across the dense and dilute phase, we calculate the largest cluster of proteins using Freud[6] Python library (version 2.12.1) with a distance criterion where protein molecules within $1.5\,\sigma_P$ are considered part of the same cluster, where $\sigma_P = 50$ Å is the diameter of the protein bead. The center of mass (COM) of the proteins in this largest cluster is calculated, and that position is regarded as the origin coordinate. From the COM, the distance of all protein beads is calculated, and the distribution of this distance is binned with a width of σ_P to generate the radial density distribution of proteins.

This radial density distribution is then fitted to a hyperbolic tangent function,[7] shown in Eq. (S5) to determine the dense phase concentration, the dilute phase concentration, and the location and width of the interface.

$$C(r) = \frac{1}{2}\big(C_{dense} + C_{dilute}\big) - \frac{1}{2}\big(C_{dense} - C_{dilute}\big) tanh\left(\frac{2(r - b_{int})}{c_{int}}\right) \tag{S5}$$

where $C(r)$ is the concentration of protein, C_{dense} is the dense phase protein concentration, C_{dilute} is the dilute phase protein concentration, b_{int} is the location of the interface and c_{int} is the width of interface.

For evaluating the protein concentration in case of multiphasic condensate formation, the radial density distribution is calculated as mentioned above but this distribution is then fitted to a combination of two hyperbolic tangent functions, shown in Eq. (S6) to determine the concentration of both the dense phases, the dilute phase, the width and the location of the two interfaces:

$$C(r) = A(r) \times switch(r) + B(r) \times \big(1 - switch(r)\big) \tag{S6}$$

where

$$A(r) = \frac{1}{2}\big(C_{dense,I} + C_{dense,II}\big) - \frac{1}{2}\big(C_{dense,I} - C_{dense,II}\big) tanh\left(\frac{2(r - b_{int,I})}{c_{int,I}}\right) \tag{S7}$$

$$B(r) = \frac{1}{2}\big(C_{dense,II} + C_{dilute}\big) - \frac{1}{2}\big(C_{dense,II} - C_{dilute}\big) tanh\left(\frac{2(r - b_{int,II})}{c_{int,II}}\right) \tag{S8}$$

and

$$switch(r) = \frac{1}{2}\left(1 - tanh\left(\frac{2(r - \frac{b_{int,I}+b_{int,II}}{2})}{b_{int,II} - b_{int,I}}\right)\right) \tag{S9}$$

Here, $C_{dense,I}$ is the first dense phase protein concentration, $C_{dense,II}$ is the second dense phase

protein concentration, C_{dilute} is the dilute phase protein concentration, $b_{int,I}$ and $b_{int,II}$ are the location of the first and second interfaces, $c_{int,I}$ and $c_{int,II}$ are the width of both interfaces.

Supplementary Figures

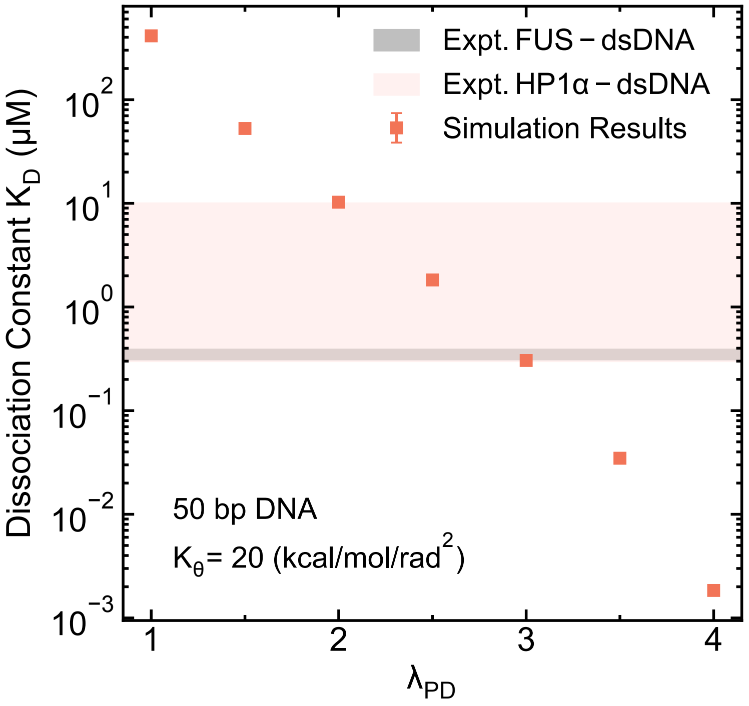

Figure S1: Dissociation constant K_D of a protein bead from a single DNA chain of length 50 bp was calculated from our simulation as a function of protein-DNA interaction strength λ_{PD}. The shaded regions highlight experimentally reported K_D values for the FUS-dsDNA[8] and HP1α-dsDNA[9] systems. The DNA bending rigidity considered in this analysis is $K_\theta = 20$ kcal/mol/rad^2.

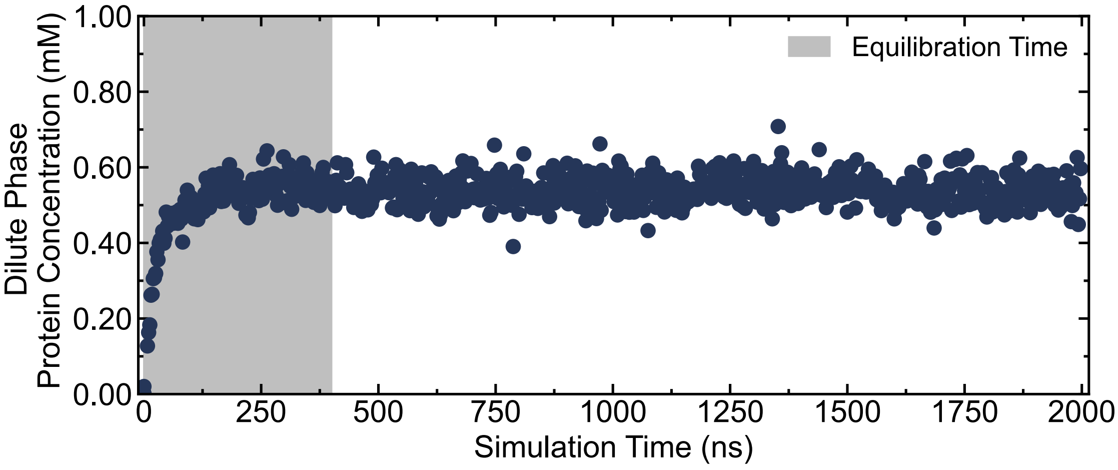

Figure S2: Dilute phase protein concentration as a function of simulation time. The initial 400 ns data (gray shaded region) was considered for equilibration period and the remaining data was used for production analysis. The DNA length considered in this analysis is 250 bp and the homotypic and heterotypic interaction strengths are $\lambda_{PP} = 3.0$ and $\lambda_{PD} = 0.3$ respectively.

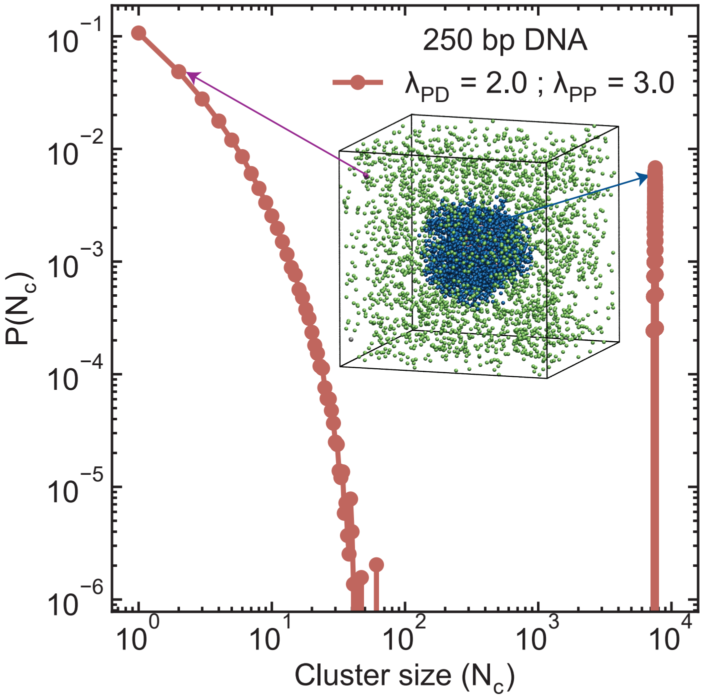

Figure S3: The probability distribution of cluster sizes for $\lambda_{PD} = 2.0$ and $\lambda_{PP} = 3.0$. The DNA length considered in this analysis is 250 bp. The inset shows a simulation snapshot highlighting the largest cluster (shown in blue color) along with smaller clusters (shown in green color).

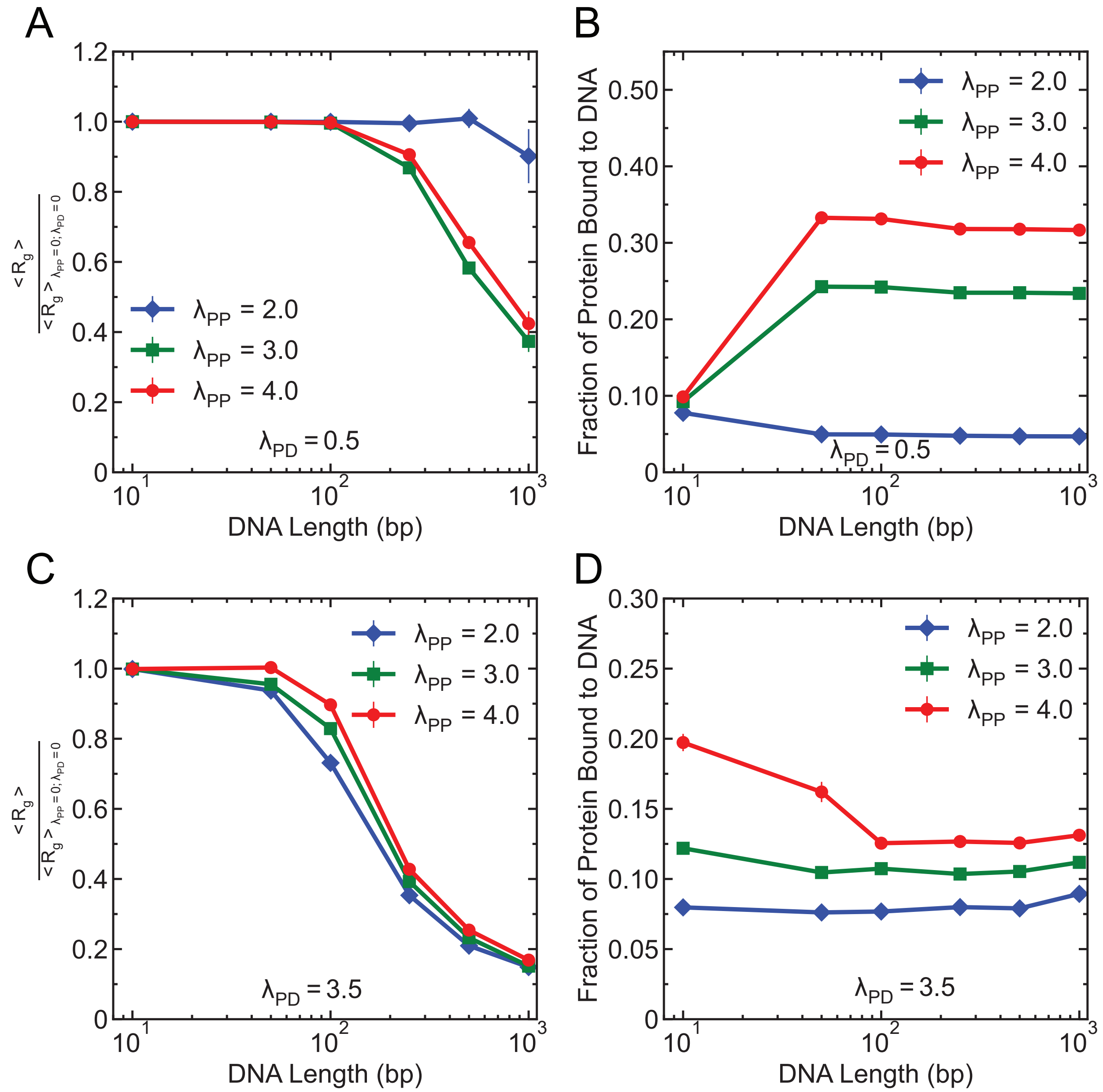

Figure S4: (A) Normalized radius of gyration (R_g) of DNA molecules as a function of DNA length for $\lambda_{PD} = 0.5$ with three different homotypic interaction strengths $\lambda_{PP} = 2.0, 3.0, 4.0$. R_g is normalized by the R_g of corresponding DNA chains in the absence of attractive interactions ($\lambda_{PP} = \lambda_{PD} = 0$). (B) Fraction of proteins bound to DNA as a function of DNA length under the same conditions as in panel (A). (C) Normalized R_g of DNA as a function of DNA chain length for $\lambda_{PD} = 3.5$ with three different homotypic strengths λ_{PP}. (D) Fraction of proteins bound to DNA as a function of DNA length under the same conditions as in panel (C).

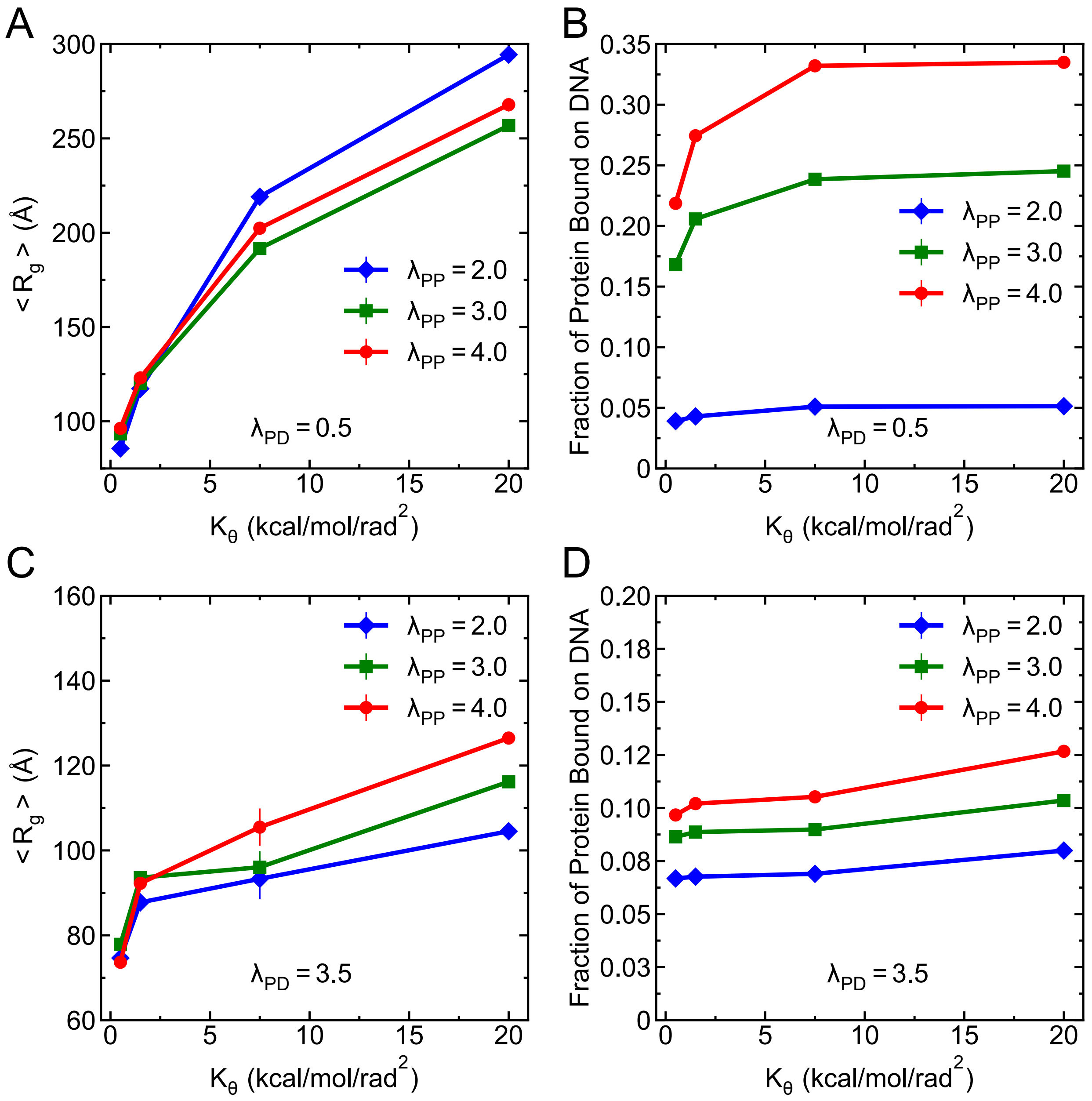

Figure S5: (A) Average R_g of DNA of length 250 bp as a function of bending rigidity (K_θ) for $\lambda_{PD} = 0.5$ with three different homotypic interaction strengths $\lambda_{PP} = 2.0, 3.0, 4.0$. (B) Fraction of proteins bound to DNA as a function of K_θ under the same conditions as in panel (A). (C) Average R_g of DNA as a function of K_θ for $\lambda_{PD} = 3.5$ with three different homotypic strengths λ_{PP}. (D) Fraction of proteins bound to DNA as a function of K_θ under the same conditions as in panel (C).

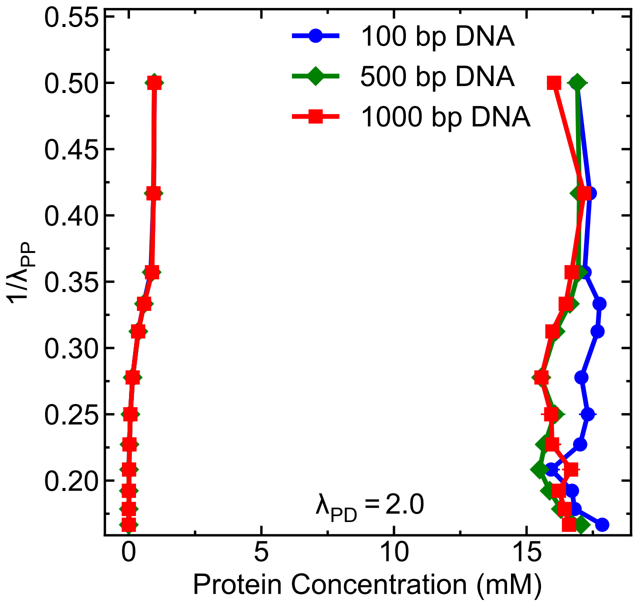

Figure S6: Phase diagram showing protein concentrations in the dilute and dense phases as a function of protein–protein interaction strength (λ_{PP}), for three different DNA lengths: 100 bp, 500 bp and 1000 bp at strong heterotypic interaction strength ($\lambda_{PD} = 2.0$).

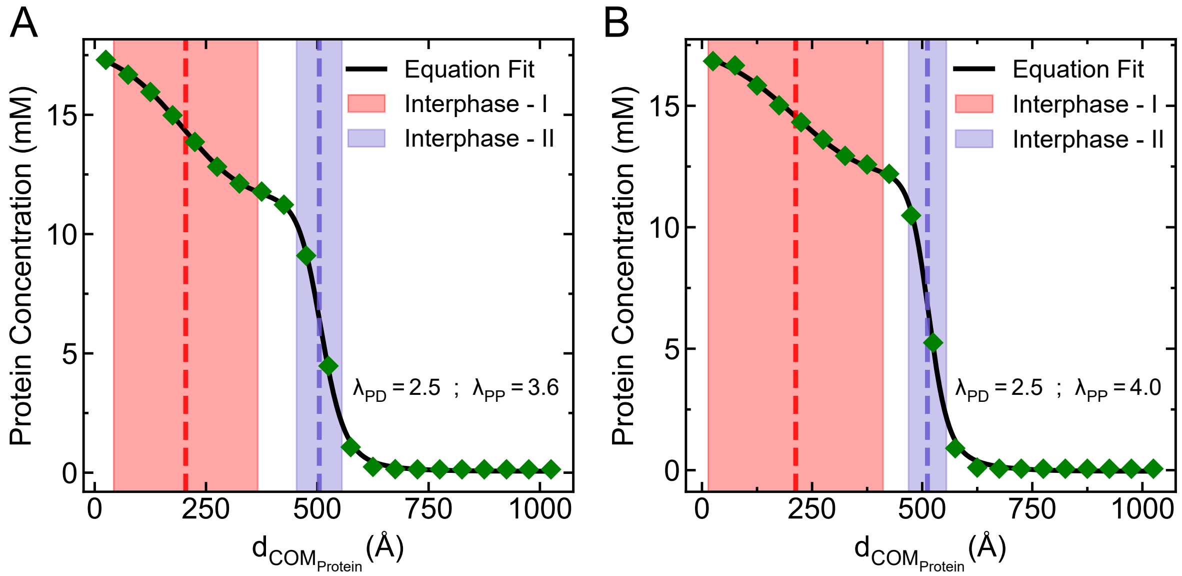

Figure S7: Radial density profile of proteins from the center of mass (COM) of proteins in the largest cluster at (A) $\lambda_{PD} = 2.5$, $\lambda_{PP} = 3.6$, and (B) $\lambda_{PD} = 2.5$, $\lambda_{PP} = 4.0$, showing two distinct protein populations. The profile is fitted with a double hyperbolic tangent function (solid black line, Eq. (S6)), capturing the presence of an inner DNA-associated protein-rich region (dense phase-I) and an outer protein-rich region not in contact with DNA (dense phase-II). Red and blue dotted lines represent the location of the two interphases between dense phase-I, dense phase-II and between dense phase-II and dilute phase, respectively. The DNA length considered in this analysis is 250 bp.

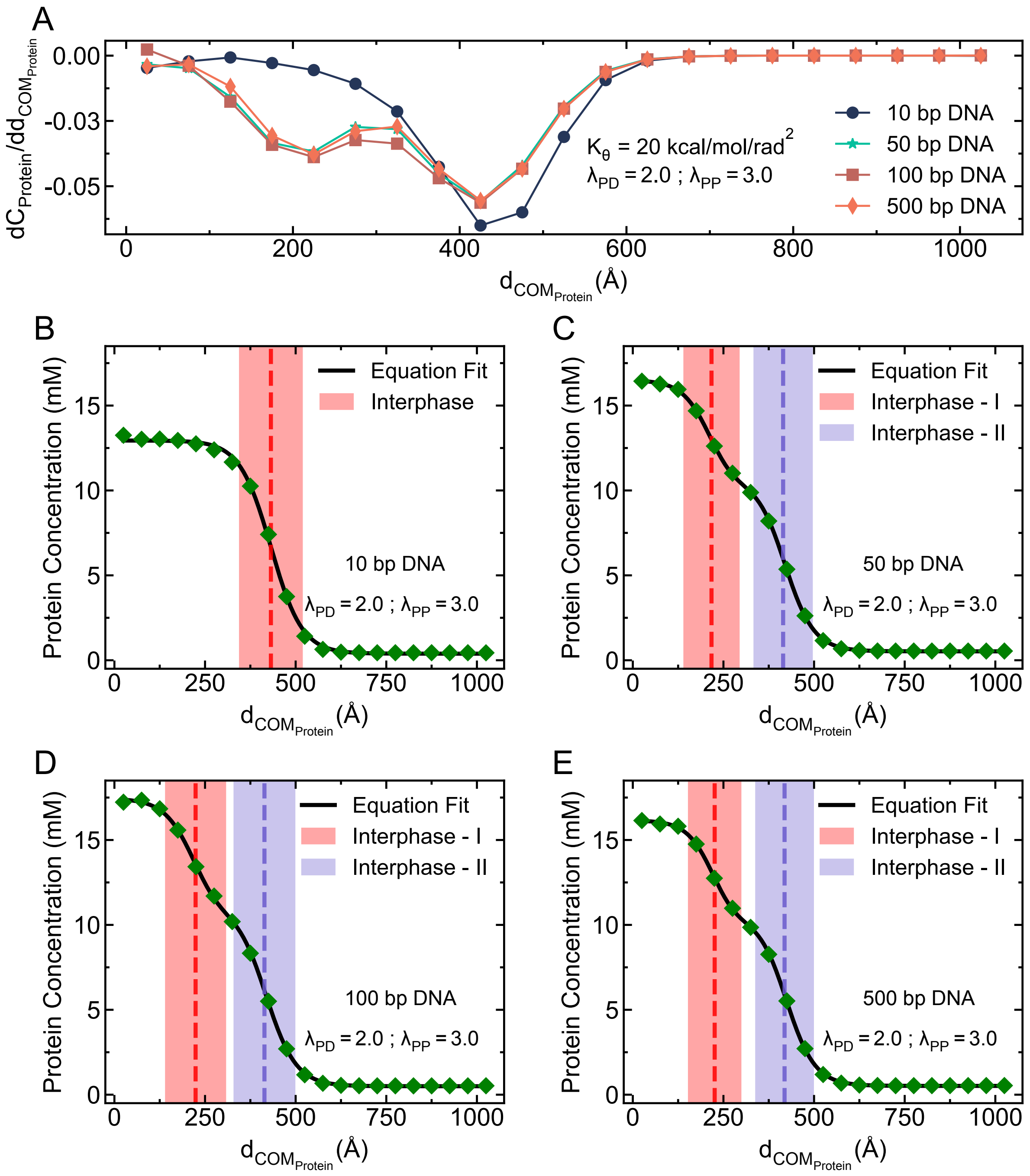

Figure S8: (A) Gradient of the protein concentration as a function of distance from the center of mass of proteins in the largest cluster, calculated for different lengths of DNA at $\lambda_{PD} = 2.0$, $\lambda_{PP} = 3.0$. Protein condensates formed with 10 bp DNA shows a single inflection point indicating the existence of a homogeneous single dense phase, whereas longer DNA chains show two inflection points indicating phase-in-phase assembly. Radial density profile of proteins from the COM of proteins in the largest cluster at $\lambda_{PD} = 2.0$, $\lambda_{PP} = 3.0$ for DNA of length (B) 10 bp, (C) 50 bp, (D) 100 bp and (E) 500 bp.

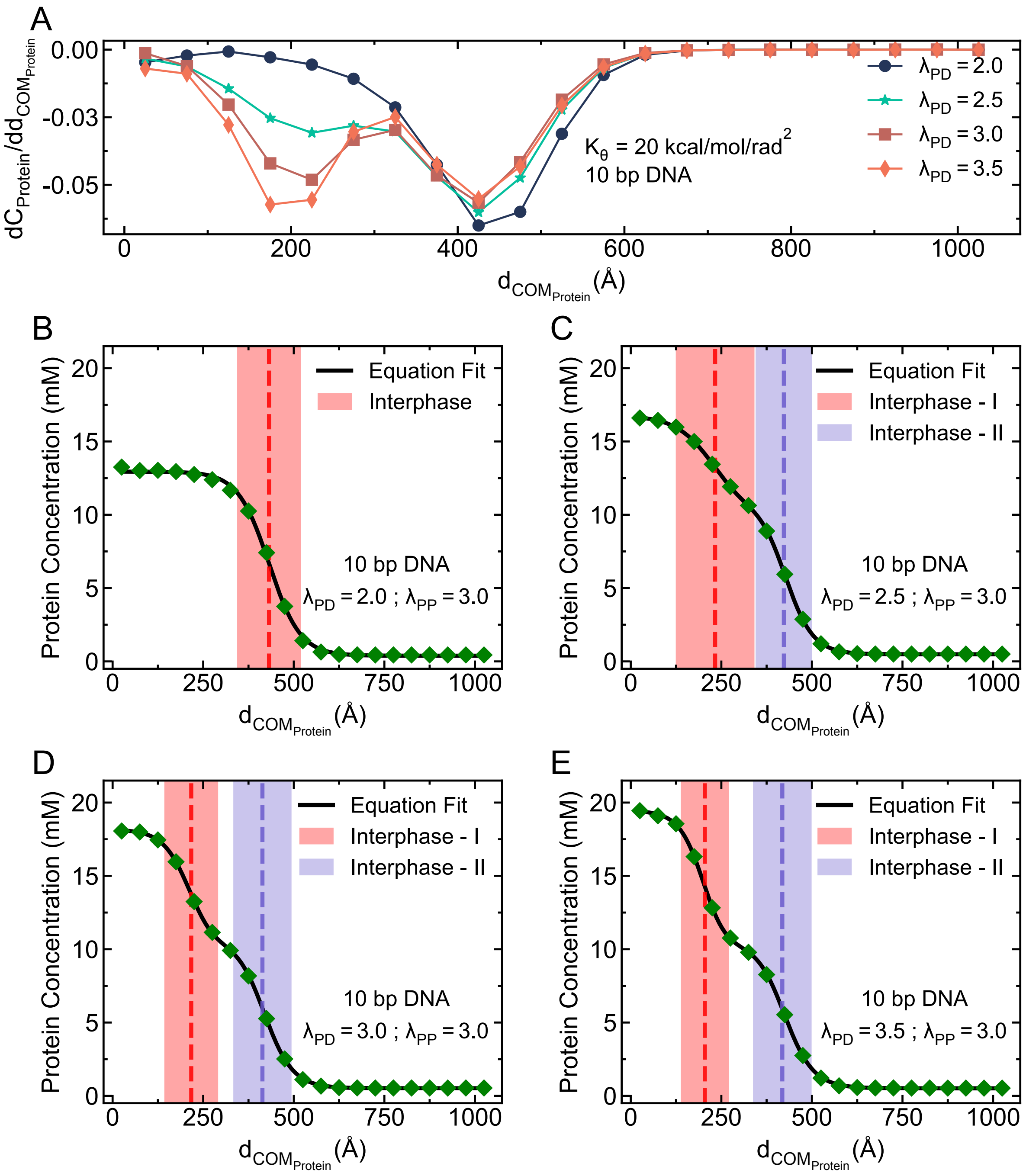

Figure S9: (A) Gradient of the protein concentration as a function of distance from the center of mass of proteins in the largest cluster, calculated for different heterotypic interaction strength for 10 bp DNA at $\lambda_{PP} = 3.0$. A single inflection point indicates the existence of a homogeneous single dense phase, whereas two inflection points indicate phase-in-phase assembly. Radial density profile of proteins from the COM of proteins in the largest cluster for protein condensates formed with 10 bp DNA at $\lambda_{PP} = 3.0$ for heterotypic interaction strength of (B) $\lambda_{PD} = 2.0$, (C) $\lambda_{PD} = 2.5$, (D) $\lambda_{PD} = 3.0$ and (E) $\lambda_{PD} = 3.5$.

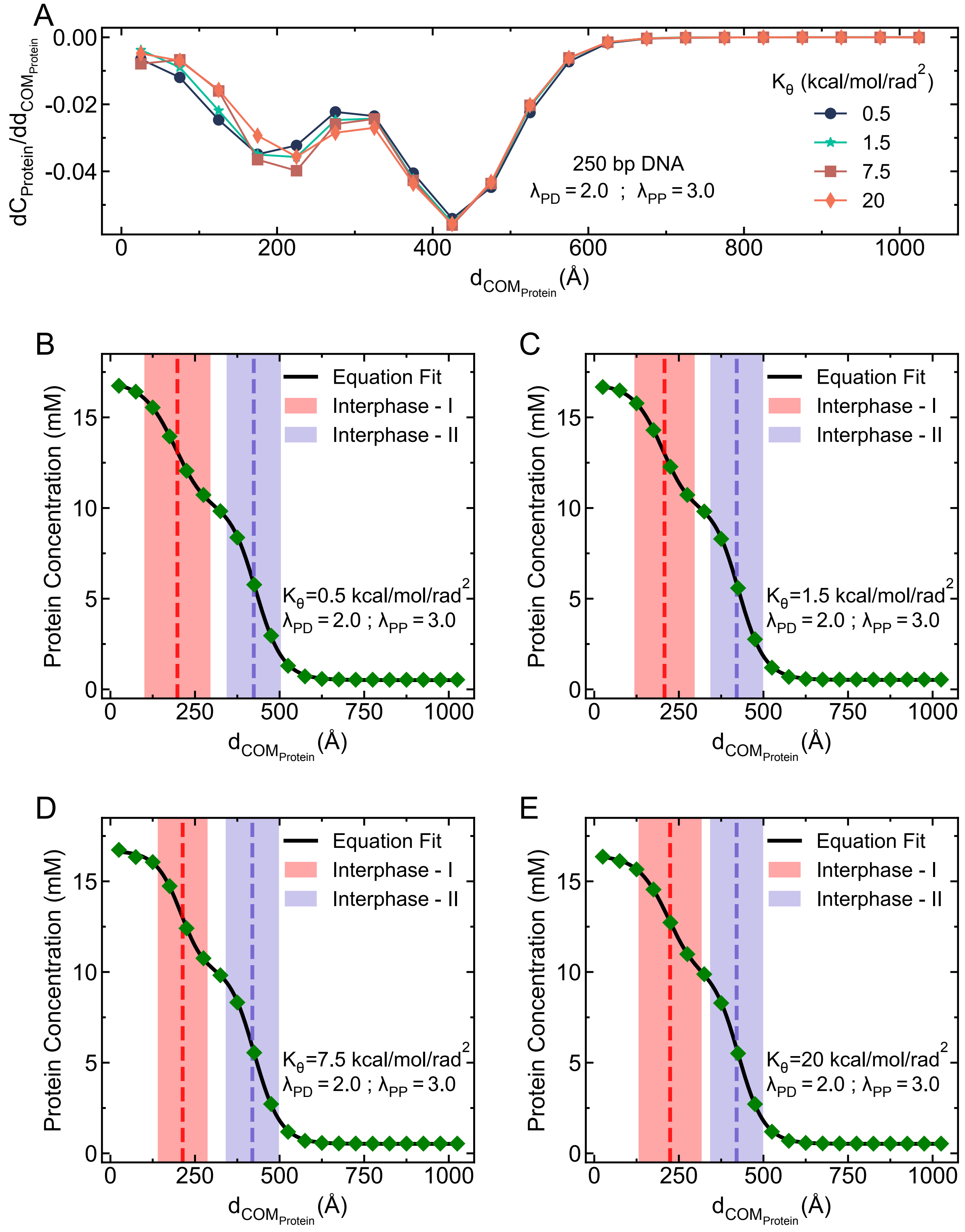

Figure S10: (A) Gradient of the protein concentration as a function of distance from the center of mass of proteins in the largest cluster, calculated for different bending rigidity for 250 bp DNA at $\lambda_{PD} = 2.0$, $\lambda_{PP} = 3.0$. Radial density profile of proteins from the COM of proteins in the largest cluster at $\lambda_{PD} = 2.0$, $\lambda_{PP} = 3.0$ for different K_θ values of (B) 0.5, (C) 1.5, (D) 7.5 and (E) 20 kcal/mol/rad^2. The DNA length considered in this analysis is 250 bp.

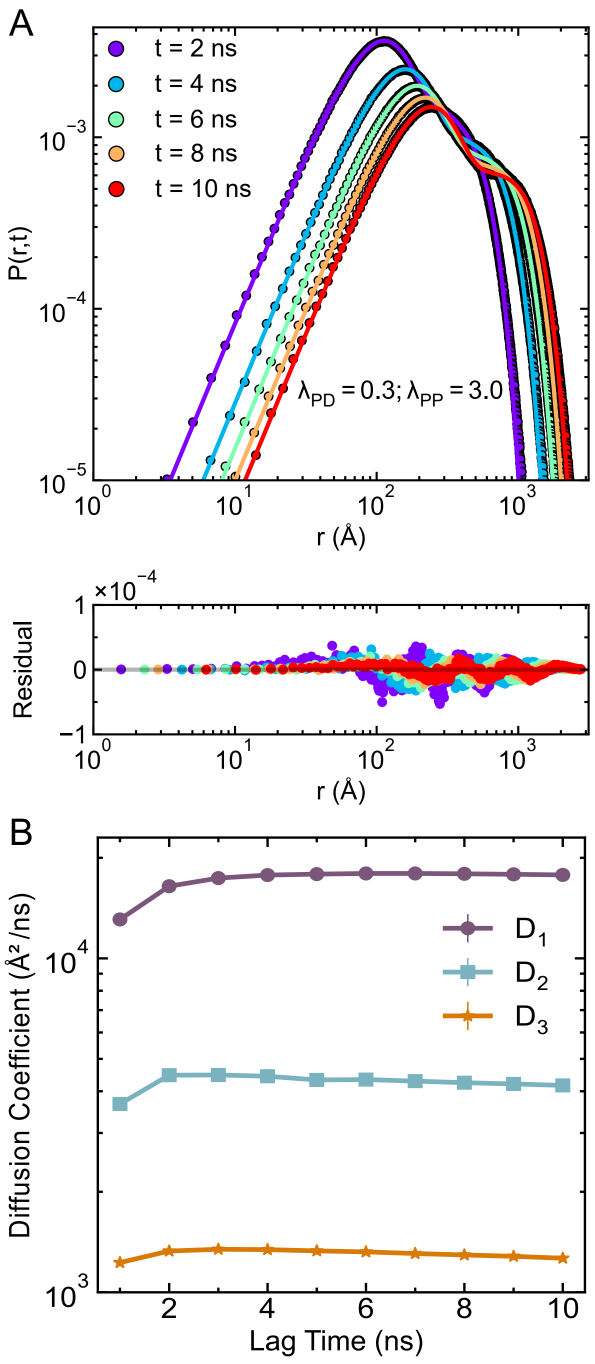

Figure S11: (A) Radial displacement probability distributions of proteins, $P(r,t)$, for different lag times t, under weak heterotypic ($\lambda_{PD} = 0.3$) and strong homotypic ($\lambda_{PP} = 3.0$) interaction strengths. The points denote histogram data from simulations, while solid lines show the best-fit using a sum of three Gaussian components (Eq. 6 in the main text). The difference between the Gaussian fitted lines and the simulation data points are represented as residuals from the data fitting for each lag time t (lower panel), confirming the quality of fit. (B) Diffusion coefficients (D_i) corresponding to the three dynamic modes extracted from the Gaussian fits in (A), shown as a function of lag time. The plateau behavior indicates that each mode exhibits stable diffusivity across lag times.

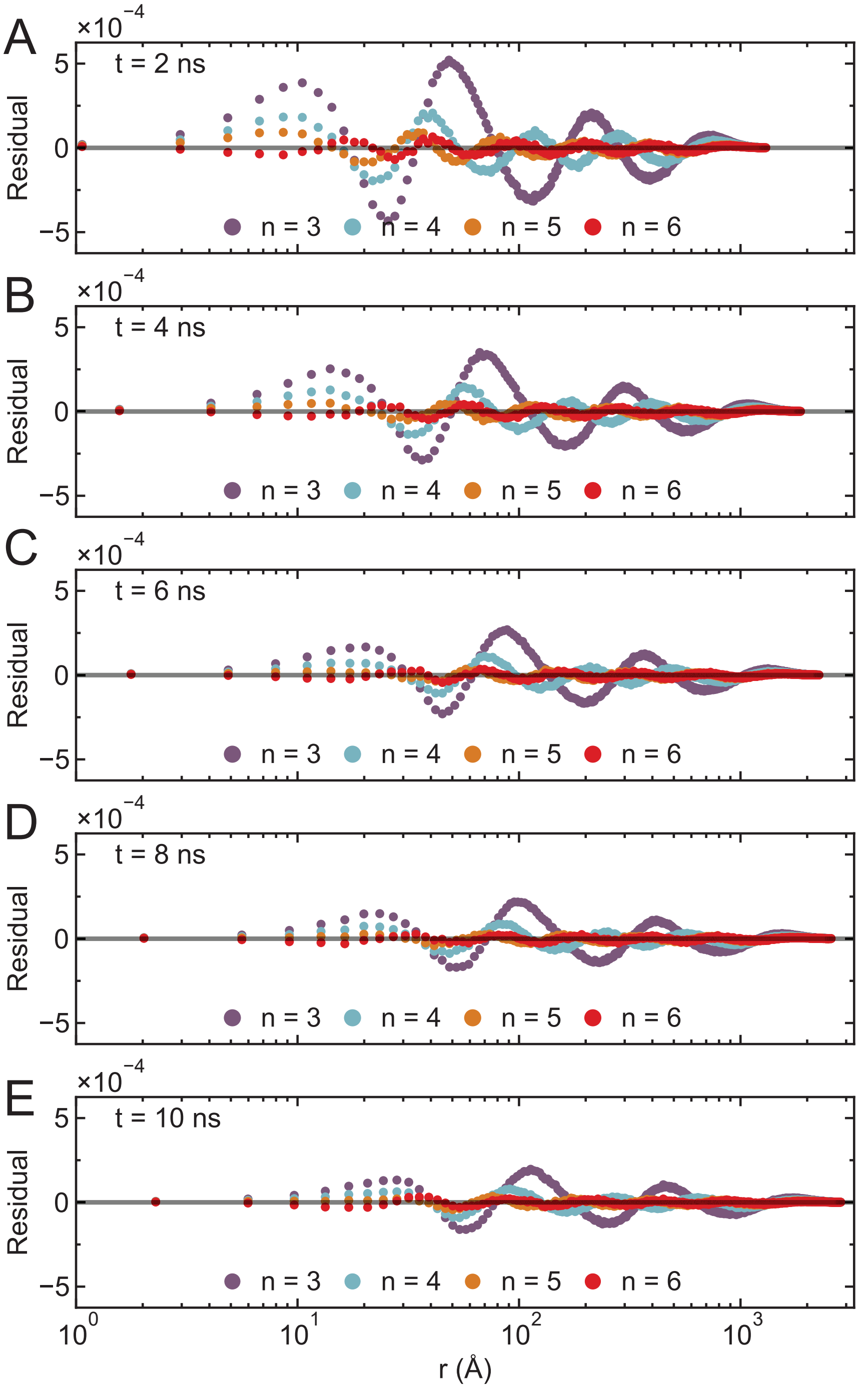

Figure S12: Residual analysis for Gaussian fitting of protein displacement distribution in multiphase condensates. To determine the optimal number of Gaussian components describing protein displacement distributions for the multiphasic condensate ($\lambda_{PD} = 2.0$ and $\lambda_{PP} = 3.0$ as considered in Fig. 7A in the main text), we calculated the residuals between simulation data and fitted curves using $n = 3$, $n = 4$, $n = 5$, and $n = 6$ Gaussian components at a fixed lag time of (A) $t = 2$ ns, (B) $t = 4$ ns, (C) $t = 6$ ns, (D) $t = 8$ ns and (E) $t = 10$ ns, as a function of radial distance.

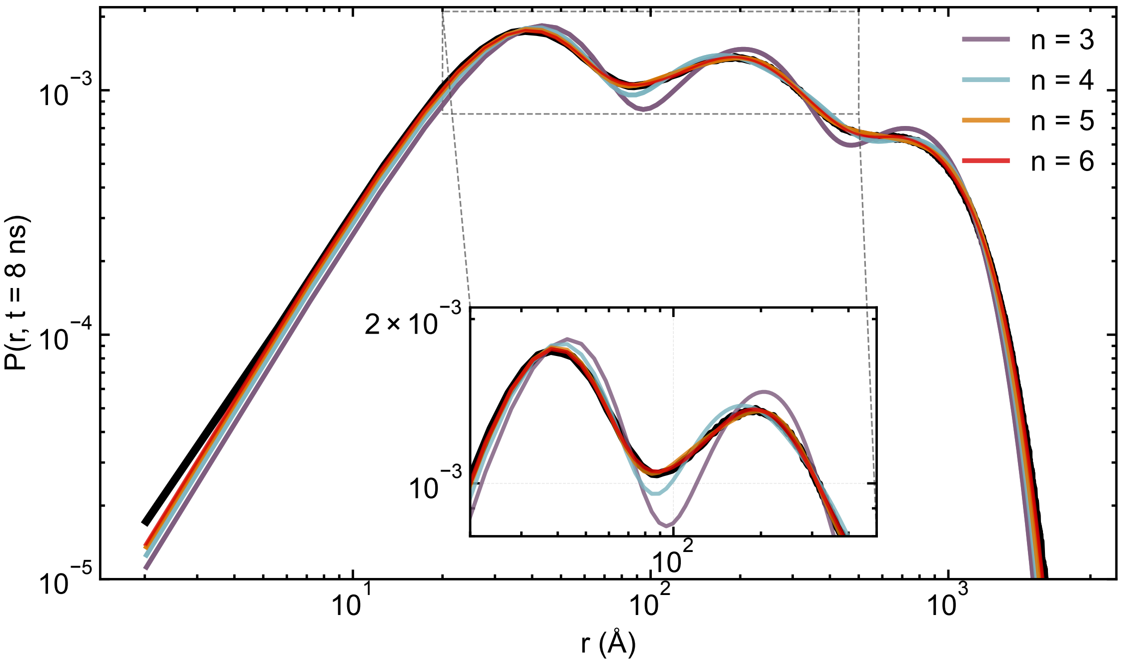

Figure S13: Gaussian fitting of protein displacement distribution in multiphase condensates to determine the optimal number of Gaussian components describing protein displacement distributions for the multiphasic condensate ($\lambda_{PD} = 2.0$ and $\lambda_{PP} = 3.0$ as considered in Fig. 7A in the main text). The black line shows the result from simulations and color lines show different Gaussian components fitting for a fixed lag time $t = 8$ ns as a function of radial distance. Both the fits from $n = 5$ and $n = 6$ Gaussian components coincide (as seen in inset), indicating that $n = 5$ is optimum for fitting the displacement distribution data.

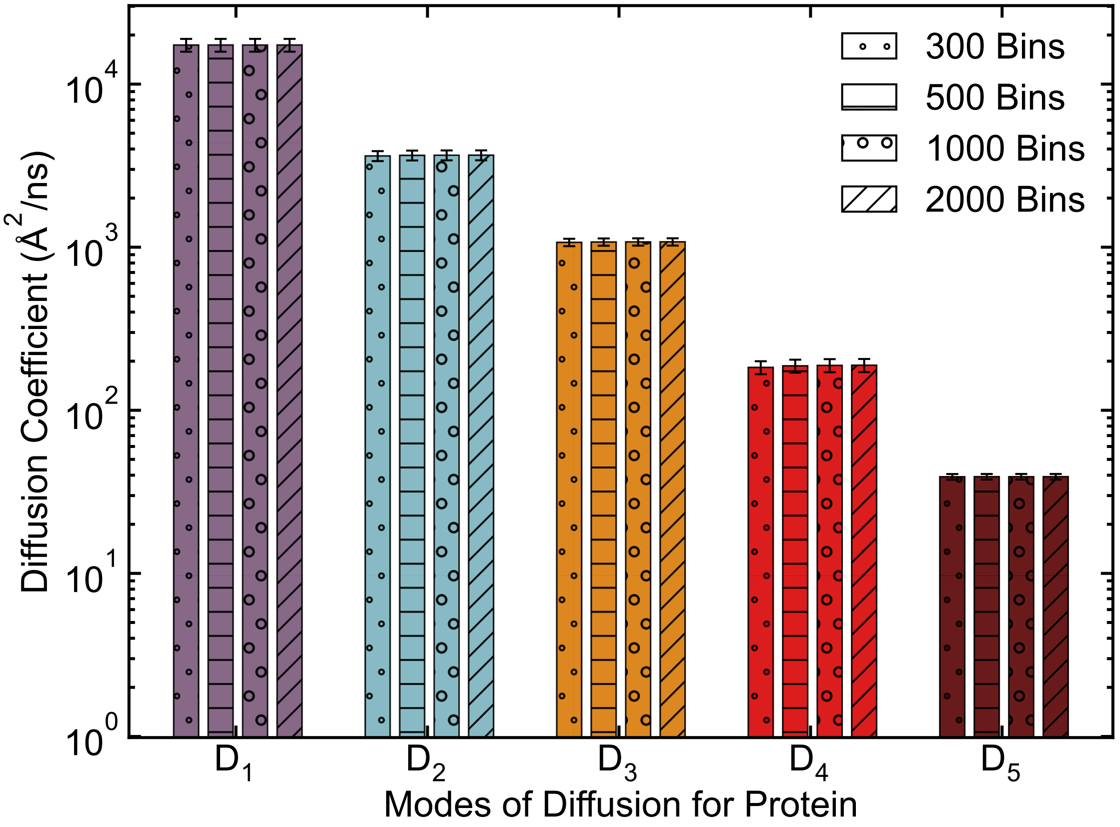

Figure S14: Comparison of the average diffusion coefficients using four different bin sizes for the displacement distribution. In all cases, we consistently recovered five different dynamic modes of protein and the fitted diffusion coefficients were unaffected by the choice of bin size.

References

(1) Jost Lopez, A.; Quoika, P. K.; Linke, M.; Hummer, G.; Köfinger, J. Quantifying Protein–Protein Interactions in Molecular Simulations. *The Journal of Physical Chemistry B* **2020**, *124*, 4673–4685.

(2) Thompson, A. P.; Aktulga, H. M.; Berger, R.; Bolintineanu, D. S.; Brown, W. M.; Crozier, P. S.; in 't Veld, P. J.; Kohlmeyer, A.; Moore, S. G.; Nguyen, T. D.; Shan, R.; Stevens, M. J.; Tranchida, J.; Trott, C.; Plimpton, S. J. LAMMPS - a flexible simulation tool for particle-based materials modeling at the atomic, meso, and continuum scales. *Comp. Phys. Comm.* **2022**, *271*, 108171.

(3) Fiorin, G.; Klein, M. L.; Hénin, J. Using collective variables to drive molecular dynamics simulations. *Molecular Physics* **2013**, *111*, 3345–3362.

(4) Michaud-Agrawal, N.; Denning, E. J.; Woolf, T. B.; Beckstein, O. MDAnalysis: a toolkit for the analysis of molecular dynamics simulations. *Journal of computational chemistry* **2011**, *32*, 2319–2327.

(5) Devarajan, D. S.; Rekhi, S.; Nikoubashman, A.; Kim, Y. C.; Howard, M. P.; Mittal, J. Effect of charge distribution on the dynamics of polyampholytic disordered proteins. *Macromolecules* **2022**, *55*, 8987–8997.

(6) Ramasubramani, V.; Dice, B. D.; Harper, E. S.; Spellings, M. P.; Anderson, J. A.; Glotzer, S. C. freud: A software suite for high throughput analysis of particle simulation data. *Computer Physics Communications* **2020**, *254*, 107275.

(7) Kuo, I. W.; Mundy, C. J.; Eggimann, B. L.; McGrath, M. J.; Siepmann, J. I.; Chen, B.; Vieceli, J.; Tobias, D. J. Structure and dynamics of the aqueous liquid-vapor interface: a comprehensive particle-based simulation study. *The Journal of Physical Chemistry B* **2006**, *110*, 3738–3746.

(8) Wang, X.; Schwartz, J. C.; Cech, T. R. Nucleic acid-binding specificity of human FUS protein. *Nucleic acids research* **2015**, *43*, 7535–7543.

(9) Keenen, M. M.; Brown, D.; Brennan, L. D.; Renger, R.; Khoo, H.; Carlson, C. R.; Huang, B.; Grill, S. W.; Narlikar, G. J.; Redding, S. HP1 proteins compact DNA into mechanically and positionally stable phase separated domains. *elife* **2021**, *10*, e64563.
